# Rapid protamine evolution suppresses meiotic drive in *Drosophila*

**DOI:** 10.1126/sciadv.aeb5514

**Published:** 2026-10-07

**Authors:** Ching-Ho Chang, Aida Flor A. de la Cruz, Hung-Yu Lai, Isabel Mejia Natividad, Alex Noyola, Elian Angelo Magsino Abellanosa, Nicolas D. Lee, Harmit S. Malik

**Affiliations:** ^1^ Division of Basic Sciences, Fred Hutchinson Cancer Center, Seattle, WA 98109, USA.; ^2^ Institute of Molecular Biology, Academia Sinica, Taipei, Taiwan.; ^3^ Molecular and Cell Biology, Taiwan International Graduate Program, Academia Sinica, Taipei, Taiwan.; ^4^ Graduate Institute of Life Sciences, National Defense Medical Center, Taipei, Taiwan.; ^5^ Howard Hughes Medical Institute, Fred Hutchinson Cancer Center, Seattle, WA 98109, USA.

## Abstract

Many animal species replace histones with protamines during spermatogenesis. Although essential for sperm function, protamines evolve rapidly for reasons that remain unknown. Using in vivo gene replacement, we examined the causes and consequences of the rapid evolution of *Mst77F*, a protamine essential for male fertility in *Drosophila melanogaster* (*D. melanogaster*). Replacing *Mst77F* with divergent orthologs caused DNA compaction defects in X-bearing sperm, resulting in fewer mature X-bearing sperm and male-biased progeny. Reducing *D. melanogaster Mst77F* dosage caused the same bias, whereas increasing the dosage of *Mst77F* orthologs rescued it. Engineered sex chromosome fusions revealed that *Mst77F* protects the *D. melanogaster* X chromosome from a killer-target meiotic drive system in a dosage- and sequence-dependent manner. Unlike in *D. melanogaster*, *Mst77F* was dispensable for fertility in *Drosophila yakuba* (*D. yakuba*) but remained important for suppressing sex ratio distortion. Our findings show that relentless pressure to suppress sex chromosome meiotic drive underlies the rapid evolution of protamines.

## INTRODUCTION

Most eukaryotes rely on histones and histone variants to package their DNA and regulate processes such as transcription, DNA repair, and chromosome segregation (*1*). However, various animal species have independently evolved distinct DNA compaction proteins, collectively referred to as protamines, to facilitate the compact packaging of genomes by up to 100-fold within sperm heads relative to somatic nuclei (*2*–*5*). For example, mammalian protamines are likely derived from linker histones (*6*, *7*), whereas *Drosophila* protamines (or sperm nuclear basic proteins) are derived from high mobility group (HMG) box encoding transcription factors (*2*, *8*). Although protamines are absent in some animals, they are essential for male fertility in all studied species that encode them, including mammals and *Drosophila* (*4*).

Despite their critical roles in sperm genome packaging and male fertility, protamine genes are among the fastest-evolving genes across various animal species, including mammals (*9*–*11*) and *Drosophila* species (*2*). A prevailing hypothesis suggests that the rapid evolution of protamines results from their role in shaping the sperm head and enhancing sperm success amid competition for fertilization (*10*, *12*, *13*). Protamine loss or mutations impair sperm head morphology and fertilization (*14*–*16*). However, there is no direct evidence that rapid protamine evolution is driven by a selective advantage for successful fertilization (*17*). Moreover, the intensity of sperm competition is either poorly correlated or not correlated with protamine evolutionary rates (*9*, *10*, *18*, *19*). Some studies have alternatively suggested that the rapid evolution of protamines in mammals results from hypermutation at their CpG-bearing arginine codons, followed by selection to restore optimal arginine content for protamine function (*20*).

Our recent phylogenomic analysis of protamine genes in *Drosophila* species, which encode at least 15 distinct protamine genes (*21*–*24*), proposed an alternative explanation for the rapid evolution of protamines (*2*). Our analysis revealed extensive turnover of protamine genes associated with sex chromosome evolution, including multiple independent duplications and amplification events on sex chromosomes across *Drosophila* species (*2*, *25*–*28*). Such gene duplicates, including X-linked paralogs of *ProtA*/*B* and Y-linked paralogs of *Mst77F* (*Mst77Y*), could evolve roles as sperm killers—selfish genes that enhance their propagation at the expense of host fertility (*25*, *26*, *29*). Conversely, in the *montium* subgroup of *Drosophila* species, half of the repertoire of ancestrally conserved protamine genes on autosomes was lost coincident with an X^Y chromosomal fusion, which obviated sex chromosome competition (*2*). These findings suggest that rapid protamine evolution may be driven by the need to suppress sex chromosome meiotic drive, i.e., the non-Mendelian overtransmission of one sex chromosome arising from conflict between X- and Y-bearing sperm, thereby maintaining male fertility and preserving the Fisherian optimal 50:50 sex ratio in resulting progeny (*30*, *31*). However, the biological causes underlying the rapid evolution of protamines remained experimentally unverified in any species.

Here, using gene knockouts, ortholog replacements, and engineered sex chromosome fusions, we show that the autosomal protamine *Mst77F* suppresses sex chromosome meiotic drive in *Drosophila melanogaster* and that its correct sequence and dosage are required for this suppression. This distortion arises from unequal maturation rates of sperm carrying X or Y chromosomes during spermatogenesis. In *Drosophila melanogaster* (*D. melanogaster*), transgenic flies with reduced *Mst77F* dosage or orthologs from other species exhibit chromatin condensation defects in sperm bearing X chromosomes, resulting in their underrepresentation in mature sperm and ultimately leading to male-skewed progeny. Using engineered sex chromosome fusions, we show that the distortion reflects a killer-target mechanism acting on X-linked sequences in *D. melanogaster*. Extending these findings to a second species, we show that knocking out *Mst77F* in *Drosophila yakuba* (*D. yakuba*) does not cause complete sterility but does cause significantly male-biased progeny. Our results suggest that the need to suppress competitive interactions between sperm with different genotypes, such as X- and Y-bearing sperm, during spermatogenesis may be one of the key forces driving the rapid evolution of protamines in *Drosophila* and likely other animal species.

## RESULTS

### *Mst77F* protamine replacements incompletely rescue male fertility in *D. melanogaster*

Using CRISPR-Cas9 and two guide RNAs (gRNAs), we generated a complete *Mst77F* knockout (*Mst77F KO*) by replacing all protein-coding sequences with an attP site, enabling subsequent site-specific ΦC31 recombinase–mediated insertion (Fig. 1A and fig. S1, A and B). Recapitulating earlier findings (*21*, *32*–*34*), we observed that *Mst77F KO* homozygous males are entirely sterile, producing no mature sperm or offspring (Fig. 1B and fig. S1C). Spermatids in *Mst77F KO* males still appear to undergo the histone-to-protamine transition, removing most histones and incorporating other protamines, e.g., *ProtA/B* (*34*). However, we observed that some spermatids have larger nuclei (fig. S1D), consistent with *Mst77F*’s proposed role in condensing genomic DNA in sperm.

**Fig. 1.**
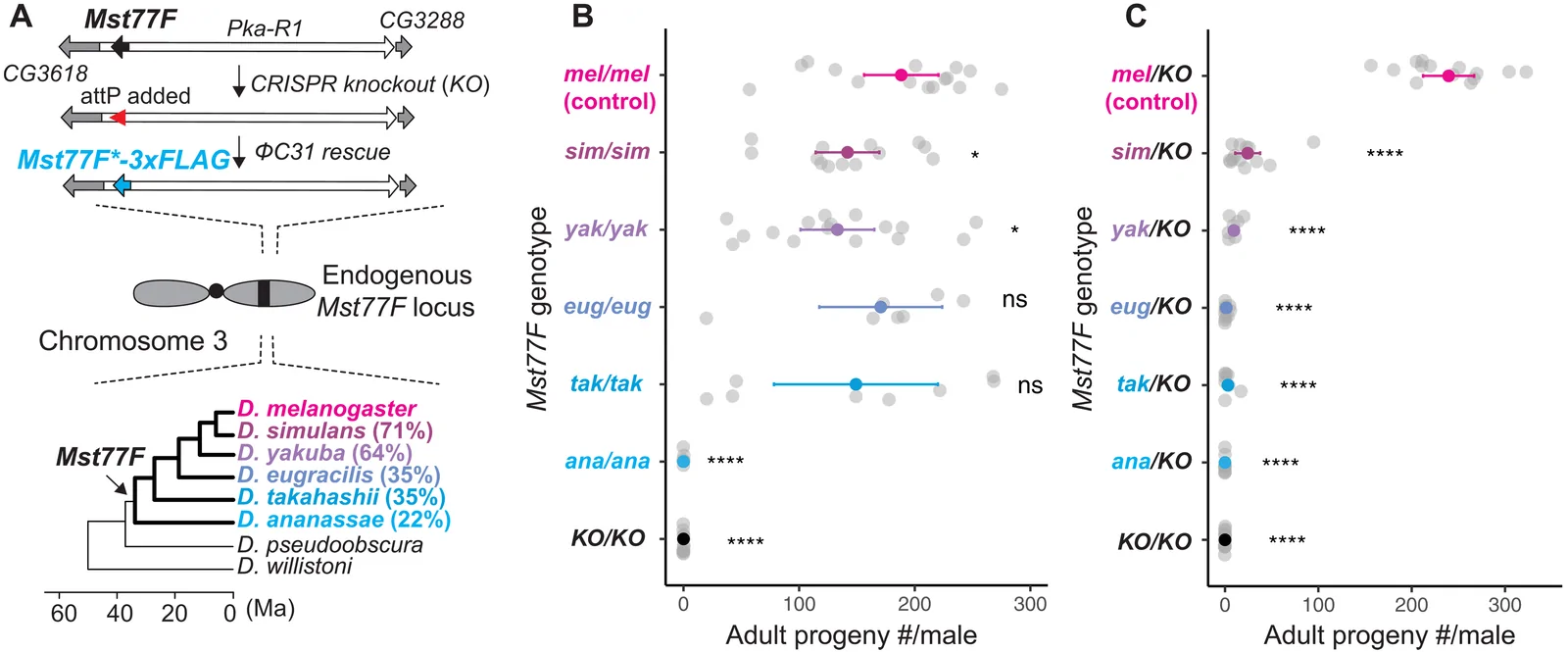
Functional divergence of the *Mst77F* essential protamine across *Drosophila* species. (**A**) Schematic of the CRISPR-Cas9–mediated knockout and ΦC31 integrase-mediated replacement of *Mst77F* in *D. melanogaster*. The endogenous *Mst77F* is located within an intron of *Pka-R1*. It was first replaced with a *DsRed* marker (not shown) and an attP site, generating male-sterile *Mst77F* knockouts (*KO*). Orthologous *Mst77F* transgenes from various *Drosophila* species were then inserted into the introduced attP site and tested for their ability to rescue male fertility. A phylogeny shows divergence of *Drosophila* species from *D. melanogaster*. Also shown is the percentage of protein identity between each ortholog and Mst77F-mel. (**B**) Comparison of the fertility of transgenic homozygous *D. melanogaster* males carrying two copies of *Mst77F* from different species. Each dot represents the total number of adult offspring produced by a single male. (**C**) Comparison of the fertility of transgenic hemizygous *D. melanogaster* males encoding only a single copy of *Mst77F* from the designated *Drosophila* species. Statistics for progeny counts were carried out using unpaired Student’s *t* tests (*P < 0.05, ****P < 0.0001, and ns, not significant).

To rescue the male sterility caused by the loss of *Mst77F*, we cloned and reintroduced the wild-type *D. melanogaster* cDNA copy of *Mst77F* (*Mst77F-mel*) using ΦC31-mediated integration at the endogenous locus (Fig. 1A). The construct included 500 bp of endogenous upstream sequence as the promoter and carried a C-terminal 3×FLAG epitope tag. As previously reported (*21*, *32*–*34*), either one or two copies of *Mst77F-mel* in the endogenous locus could rescue the *Mst77F* knockout, with each male capable of producing more than 100 offspring (Fig. 1, B and C, fig. S1C, and data S2). This shows that loss of *Mst77F* causes male sterility and that the 3×FLAG tag does not interfere with its essential function in male fertility. Immunostaining with a FLAG antibody revealed that transgenic Mst77F is incorporated into spermatid chromatin during the late canoe stage of spermatogenesis (fig. S2), like endogenous Mst77F (*34*).

On the basis of phylogenetic evidence, we inferred that *Mst77F* is one of the youngest *Drosophila* protamine genes, likely originating in the common ancestor of *D. melanogaster* and *Drosophila ananassae* ∼20 million years ago (Ma; Fig. 1A) (*35*). If the rapid evolution of *Mst77F* were tied to its roles in male fertility, we hypothesized that orthologs from various species might be maladapted for one or more of these roles in *D. melanogaster*, potentially revealing the selective pressures driving *Mst77F*’s rapid divergence. Therefore, we used the attP site introduced at the endogenous *Mst77F* locus to introduce an allelic series of C-terminal 3×FLAG-tagged *Mst77F* orthologs from five divergent *Drosophila* species encoding proteins with increasing amino acid divergence (Fig. 1A and data S1): *Drosophila simulans* (2.5 Ma, *Mst77F-sim*, 71% amino acid identity), *D. yakuba* (6 Ma, *Mst77F-yak*, 64%), *Drosophila eugracilis* (10 Ma, *Mst77F-eug*, 35%), *Drosophila takahashii* (15 Ma, *Mst77F-tak*, 35%), and *D. ananassae* (25 Ma, *Mst77F-ana*, 22%). We only swapped the protein-coding sequences, retaining the regulatory sequences from the *D. melanogaster Mst77F* locus. We confirmed that all *Mst77F* transgenes were expressed at similar stages of spermatogenesis and comparable levels to the FLAG-tagged *Mst77F-mel* transgene using immunostaining (fig. S2A) and transcriptomic analyses (fig. S3A). Subsequent Western blot analyses (fig. S3, B and C, and data S3), with or without calf intestinal alkaline phosphatase (CIP) treatment, revealed that all transgenic Mst77F proteins, except Mst77F-ana, are phosphorylated in *D. melanogaster* (fig. S3B). Consistent with normal completion of the histone-to-protamine transition, we confirmed that the histone variant His2Av is correctly removed in sperm from transgenic flies, whereas the centromeric histone Cid is appropriately retained (fig. S2B), as previously reported (*36*).

We first assessed whether *Mst77F* ortholog replacements affected male fertility in *D. melanogaster.* We found that *D. melanogaster* males homozygous for *Mst77F-ana* were sterile, like the *Mst77F-mel* knockout (Fig. 1B and fig. S1C). In contrast, transgenic males homozygous for other *Mst77F* orthologs had only a modest decrease in fertility at 25°C (Fig. 1B) or at 29°C (fig. S4A), a temperature known to perturb sperm development in *Drosophila* (*37*). However, we observed a slight but consistent decrease in seminal vesicle size among transgenic males homozygous for heterospecific *Mst77F* orthologs compared to *Mst77F-mel* homozygous males (fig. S4B and data S4). Thus, our findings show that most homozygous *Mst77F* orthologs from closely related species can complement the crucial male fertility function in *D. melanogaster*, despite sharing only 35 to 71% protein identity. Only Mst77F-ana, which is 22% identical to Mst77F-mel, cannot. Since Mst77F-ana is also the only Mst77F protein that is not phosphorylated in *D. melanogaster*, our findings suggest that phosphorylation may be a critical posttranslational requirement for *Mst77F* to fulfill its essential role in spermiogenesis (*32*).

We next reduced *Mst77F* dosage by generating hemizygous males with one *Mst77F* allele and one knockout allele. Whereas hemizygous (single-copy) *Mst77F-mel* could restore wild-type male fertility, the hemizygous *Mst77F-sim*, *Mst77F-yak*, *Mst77F-eug*, and *Mst77F-tak D. melanogaster* strains showed more than a 10-fold reduction in fertility (Fig. 1C). Thus, *Mst77F* orthologs from closely related species are haploinsufficient for normal male fertility in *D. melanogaster*, but this impairment can be relieved by increased dosage (Fig. 1B).

### *Mst77F* protamine replacements unleash sex ratio distortion in *D. melanogaster*

We previously hypothesized that autosome-encoded protamines, such as *Mst77F*, might evolve rapidly to suppress competition between X- and Y-bearing sperm, thereby ensuring optimal sex ratios and high male fertility (*2*). To test this hypothesis, we evaluated how the *Mst77F* gene replacements affected the sex ratio of the resulting adult progeny. We crossed transgenic *D. melanogaster* (XY) males encoding two copies of *Mst77F-mel* to wild-type *D. melanogaster* females (Fig. 2A). These crosses produced ∼50% males at both 25°C (Fig. 2, B and C) and 29°C (fig. S4A), consistent with Mendelian expectations, indicating equal success of X- and Y-bearing sperm.

**Fig. 2.**
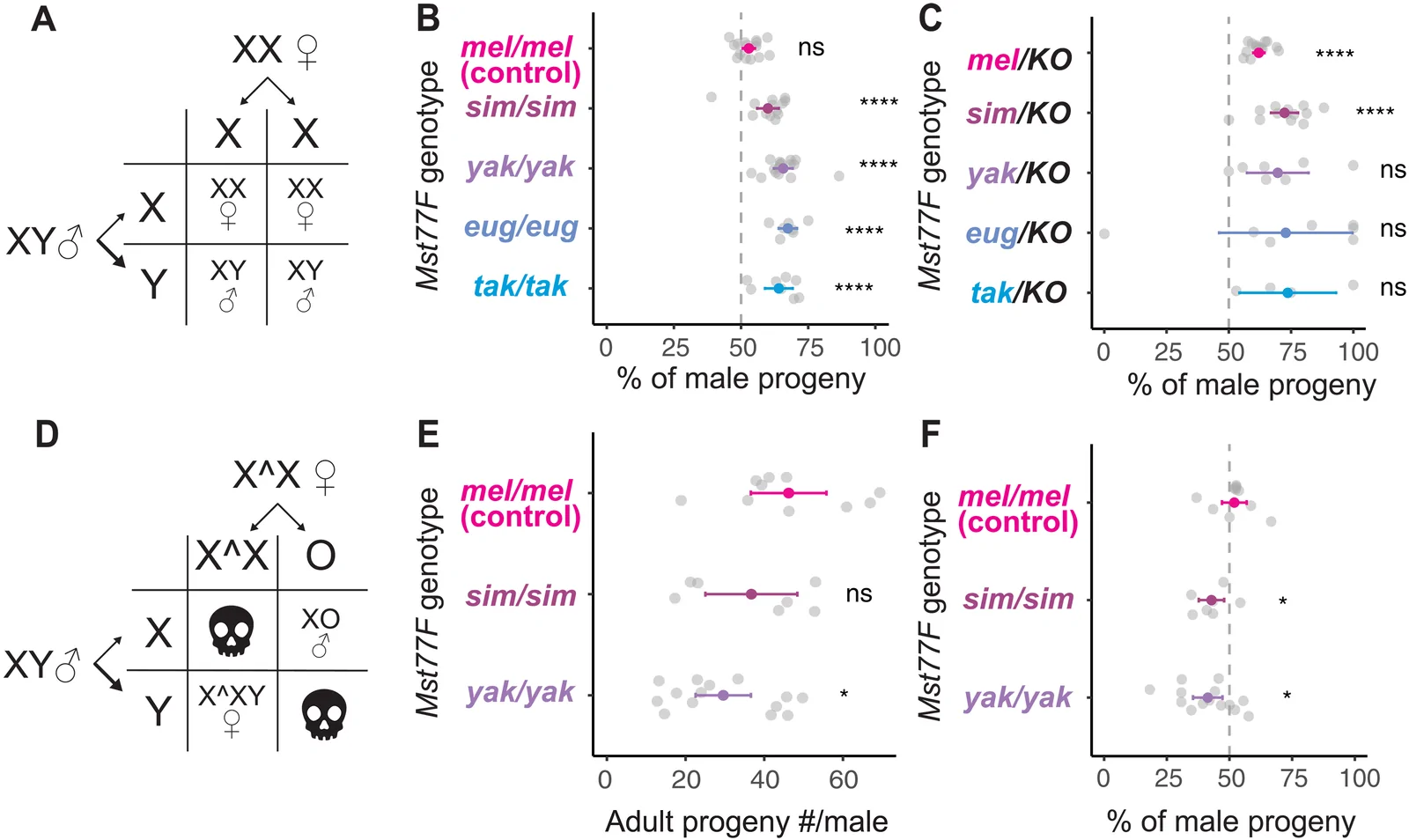
*Mst77F* gene replacements in *D. melanogaster* generate progeny with a biased sex ratio. (**A**) A Punnett square illustrates the expected genotypes from crosses between transgenic *Mst77F*-encoding XY males and wild-type XX females. Sex ratios of resulting adult progeny from transgenic *D. melanogaster* males carrying (**B**) two copies of *Mst77F* from the designated species or (**C**) one copy of *Mst77F* from the designated species. Each dot represents data from a single male (*n* ≥ 7). The dashed line indicates the expected 50:50 Mendelian ratio. In some cases, low fertility (<100 offspring per male) of hemizygous males precludes robust tests against a standard 50:50 ratio using χ^2^ test. (**D**) A Punnett square illustrates the expected genotypes from crosses between transgenic *Mst77F*-encoding XY males and females carrying an attached X chromosome (X^X). In matings with X^X females, fertilization by X-bearing sperm produces XO males, whereas fertilization by Y-bearing sperm produces X^XY females. Embryos carrying zero or three X chromosomes are inviable. (**E**) In crosses with X^X females, transgenic homozygous *D. melanogaster* males carrying two copies of *Mst77F-mel* have higher fertility than *D. melanogaster* males carrying two copies of *Mst77F-sim* or *Mst77F-yak.* (**F**) Transgenic homozygous *D. melanogaster* males carrying two copies of *Mst77F-mel* produce progeny with expected 50:50 Mendelian ratios. In contrast, *D. melanogaster* males carrying two copies of *Mst77F-sim* or *Mst77F-yak* produce more female-biased progeny. Statistics for deviations from the expected 50% male progeny ratio were carried out using χ^2^ tests and statistical comparisons of transgenic flies to transgenic flies encoding homozygous *Mst77F- mel* (control) using unpaired Student’s *t* tests (*P < 0.05 and ****P < 0.0001).

In contrast, fertile males with two copies of *Mst77F* orthologs, even from closely related species, produced more than 60% male offspring. The sex ratio distortion was further exacerbated by halving the dosage of *Mst77F*. *D. melanogaster* males carrying a single copy of *Mst77F-mel* produced 62% male progeny (Fig. 2C), although these males appear to be highly fecund (Fig. 1C). Males carrying only a single copy of *Mst77F* orthologs from other species yielded ∼70% male progeny (Fig. 2C). Conversely, both the fertility defects and the sex ratio distortion were alleviated by increasing the dosage of heterospecific *Mst77F-yak* (fig. S5, A and B). Moreover, heterozygous males carrying one copy of *Mst77F-mel* along with a single copy of an *Mst77F* ortholog performed better than *Mst77F-mel* hemizygous males. Thus, unlike *Mst77F-mel*, heterospecific *Mst77F* orthologs cannot fully rescue male fertility or suppress sex ratio distortion, but they do not act as dominant negatives in *D. melanogaster* (fig. S5, C and D). These findings suggest that both protein divergence and gene dosage of the *Mst77F* orthologs affect their ability to suppress sex ratio distortion.

Sex ratio distortion can arise from differential production or fertilization success of X- or Y-bearing sperm or from viability differences between female or male offspring. To differentiate between these possibilities, we crossed *Mst77F-mel*, *Mst77F-sim*, or *Mst77F-yak* homozygous male (XY) flies to females with attached-X (or X^X) chromosomes (Fig. 2D). In this cross, viable progeny can only result from nullo-X eggs fertilized by X-bearing sperm, which develop into XO males, or X^X eggs fertilized by Y-bearing sperm, which develop into X^XY females (*Drosophila* sex is determined by the X: autosome ratio, rather than the Y chromosome). This system effectively reverses the inheritance patterns of the X and Y chromosomes, allowing us to resolve confounding factors such as postzygotic female-specific lethality. We observed slightly lower fertility among transgenic males homozygous for heterospecific *Mst77F* orthologs compared to *Mst77F-mel* homozygous males (Fig. 2E). We found no skew in progeny sex ratios in crosses between *Mst77F-mel* homozygous males and X^X females (Fig. 2F). In contrast, we noted a higher proportion of X^XY females among the progeny from crosses of *Mst77F-sim* or *Mst77F-yak* homozygous males with X^X females compared to *Mst77F-mel* homozygous males (Fig. 2F). Thus, both crosses to XX and X^X females produced fewer progeny derived from X-bearing sperm. Because X-bearing sperm give rise to female progeny in the standard cross but to XO male progeny in the attached-X cross, this consistent deficit cannot be explained by sex-specific offspring viability. Instead, an overrepresentation of Y-bearing sperm relative to X-bearing sperm must underlie this skew, occurring either during spermatogenesis or at fertilization.

To test whether suppression of sex chromosome meiotic drive is a general property of *Drosophila* protamines or specific to *Mst77F*, we examined flies deleted for the protamines *ProtA* and *ProtB*, which have previously been implicated in suppressing the autosomal meiotic driver *Segregation Distorter* (*SD*) (*38*). Unlike perturbations of *Mst77F*, deletion of *ProtA* and *ProtB* did not skew progeny sex ratios (*n* = 2918 from 10 individual males; χ^2^ test, *P* > 0.9; data S2). This indicates that suppression of sex chromosome drive is not shared by all protamines, but it raises the possibility that different protamines may have evolved to counteract distinct meiotic drivers.

### Multiple domains of *Mst77F* contribute to its fertility and drive suppression functions

*Mst77F-yak* is impaired for both fertility-essential and meiotic drive suppressor functions in *D. melanogaster* (Fig. 3A), although it is separated from *Mst77F-mel* by merely 6 Ma. Both Mst77F-mel and Mst77F-yak proteins are of similar length (215 and 219 amino acid residues, respectively) but differ by 78 amino acids, highlighting the remarkably rapid sequence evolution of *Mst77F*. These amino acid differences are distributed throughout the proteins, except for the relatively conserved HMG domains and predicted nuclear localization signals (NLS; Fig. 3A). To identify the domains associated with fertility-essential or meiotic drive suppressor functions, we constructed chimeric proteins comprising different combinations of four segments of Mst77F-mel and Mst77F-yak (segments numbered 1 to 4 from N to C terminus; Fig. 3A).

**Fig. 3.**
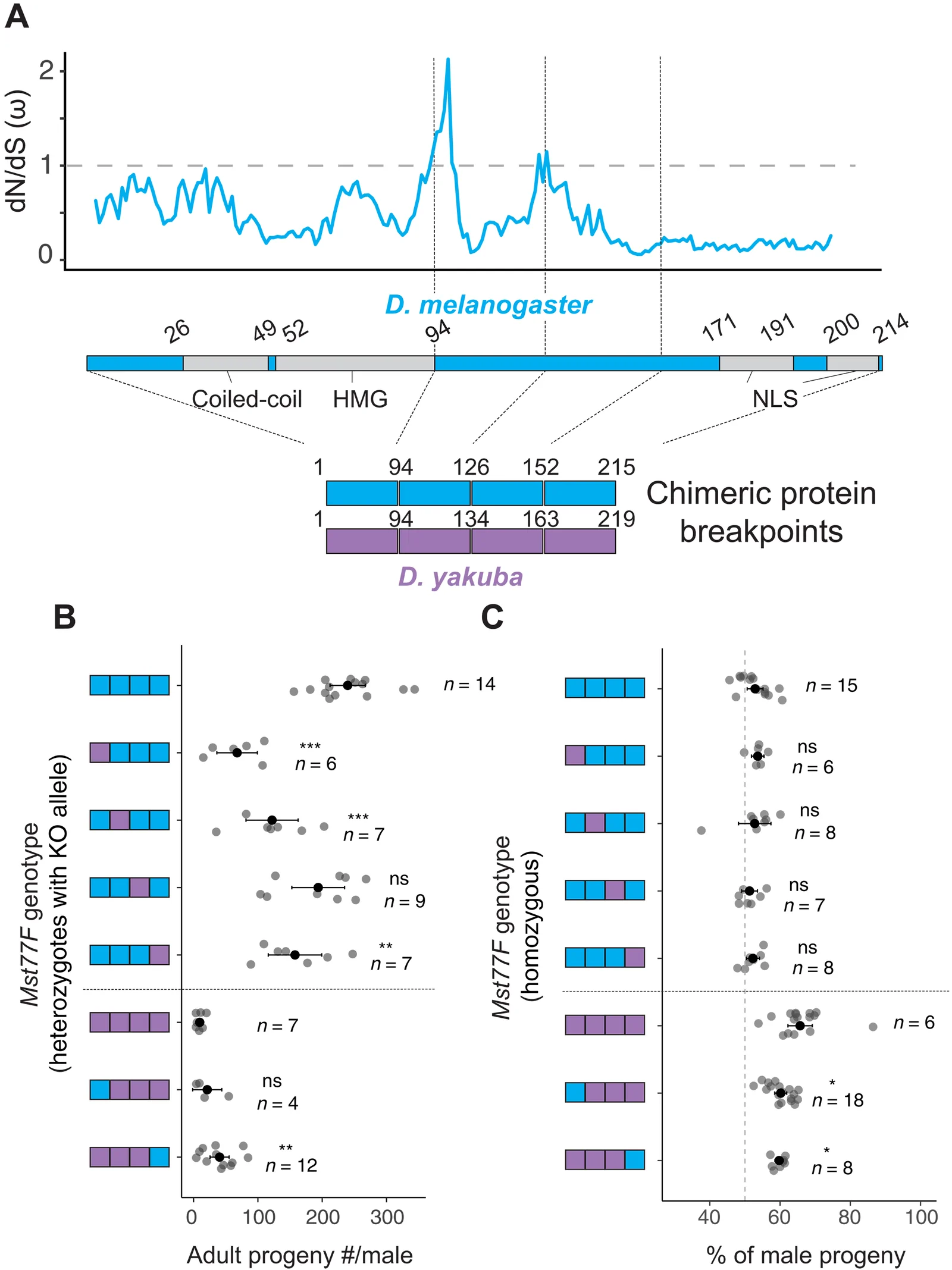
Fertility and progeny sex ratios of *D. melanogaster*–*D. yakuba Mst77F* chimeras. (**A**) Schematic representation of predicted protein domain structures of Mst77F-mel (*2*, *54*), including a coiled-coil domain (amino acids 26 to 49), a HMG box DNA binding domain (amino acids 52 to 94), and two putative NLS (amino acids 171 to 191 and 200 to 214). The top panel shows a sliding window analysis of dN/dS (ω) across the Mst77F coding sequence, highlighting a peak of positive selection (dN/dS > 1) just downstream of the HMG box. Numbers indicate the positions of three breakpoints used to generate chimeric proteins between Mst77F-mel and Mst77F-yak. Colors in the chimeric bars represent the species’ origin of the Mst77F segment, with *D. melanogaster* in blue and *D. yakuba* in magenta. (**B**) We measured the fertility of hemizygous *D. melanogaster* transgenic males expressing a single copy of *Mst77F-mel*, *Mst77F-yak*, or chimeric genes. Each dot represents data from an individual male (the number of males tested is also reported). (**C**) We measured the adult progeny sex ratio of *D. melanogaster* transgenic males expressing two copies of *Mst77F-mel*, *Mst77F-yak*, or chimeric genes. The number of males tested is also reported. Statistics for deviations from the expected 50% male progeny ratio were carried out using χ^2^ tests, and statistical comparisons of transgenic flies encoding chimeric genes were carried out to transgenic flies encoding either *Mst77F- mel* (above dashed line) or *Mst77F-yak* (below dashed line) using unpaired Student’s *t* tests (*P < 0.05, **P < 0.01, and ***P < 0.001).

Single hemizygous *Mst77F-mel* males produced more than 200 offspring on average, whereas hemizygous *Mst77F-yak* males produced fewer than 20 progeny (Fig. 1). Therefore, to provide the most sensitive assay for male fertility, we tested each chimeric *Mst77F* gene in a single copy. Swapping the first, second, or fourth segment from *Mst77F-yak* into *Mst77F-mel* resulted in a significant reduction in fertility, whereas swapping in the third segment did not affect fertility. We also conducted a reciprocal analysis and found that the first or fourth segment from *Mst77F-mel* could only marginally rescue the low fertility of *Mst77F-yak* males (Fig. 3B). Hence, optimal fertility requires multiple domains of *Mst77F-mel* (Fig. 3B).

We next investigated the *Mst77F* chimeras for their ability to suppress sex ratio distortion. *Mst77F-mel* homozygous males show no sex ratio distortion, whereas *Mst77F-yak* homozygous males yield ∼65% male progeny (Fig. 3C). Thus, to enhance the sensitivity of our comparison, we tested all chimeras as homozygotes, which also provided the added benefit of relatively higher overall fertility. We found that no single segment from *Mst77F-yak* significantly impaired *Mst77F-mel*’s capacity to suppress sex ratio distortion (Fig. 3C). Conversely, introducing either the first or the fourth segment from *Mst77F-mel* into *Mst77F-yak* significantly alleviated but did not eliminate sex ratio distortion (Fig. 3C). These results confirm that multiple segments of *Mst77F-mel* contribute to suppressing sex ratio distortion.

By comparing all seven *Mst77F* transgenes, including *Mst77F-mel*, *Mst77F-yak*, and their chimeras (*n* = 7), we identified a strong anticorrelation between male fertility and sex ratio distortion (Pearson’s *R* = −0.82, *P* = 0.01; Spearman’s rank correlation ρ = −0.857, *P* = 0.01). This anticorrelation suggests that the depletion of X-bearing sperm contributes to both sex ratio distortion and decreased male fertility.

### X chromosome–specific DNA condensation defects in spermatids underlie the sex ratio distortion in *Mst77F* replacement males

To investigate whether X- and Y-bearing gametes progress similarly through spermatogenesis, we used fluorescence in situ hybridization (FISH) to detect satellite DNA located on either Y chromosomes (*AATAC*) or autosomes (*Prod-sat*) (Fig. 4A). This strategy allowed us to distinguish X-bearing mature sperm, which hybridize solely with the *Prod-sat* probe, from Y-bearing mature sperm, which hybridize with both the *Prod-sat* and *AATAC* probes. We then calculated the proportions of X- and Y-bearing mature sperm in transgenic *D. melanogaster* males encoding two copies of different *Mst77F* orthologs. We found no X-versus-Y skew in mature sperm from *Mst77F-mel* homozygous males (Fig. 4, B and C), consistent with no observed sex ratio distortion in progeny from these males (Fig. 2). In contrast, we identified a significant overrepresentation of Y-bearing sperm in hemizygous *Mst77F-mel* males encoding a single copy of *Mst77F-mel* or in *Mst77F-sim*, *Mst77F-yak*, *Mst77F-eug*, or *Mst77F-tak* homozygous males (Fig. 4, B and C, fig. S6A, and data S5), corresponding with the sex ratio bias observed in their resulting adult progeny (Fig. 2). These findings suggest that the underrepresentation of X-bearing sperm during spermatogenesis is sufficient to explain the sex ratio distortion observed in the progeny of *Mst77F* replacement males.

**Fig. 4.**
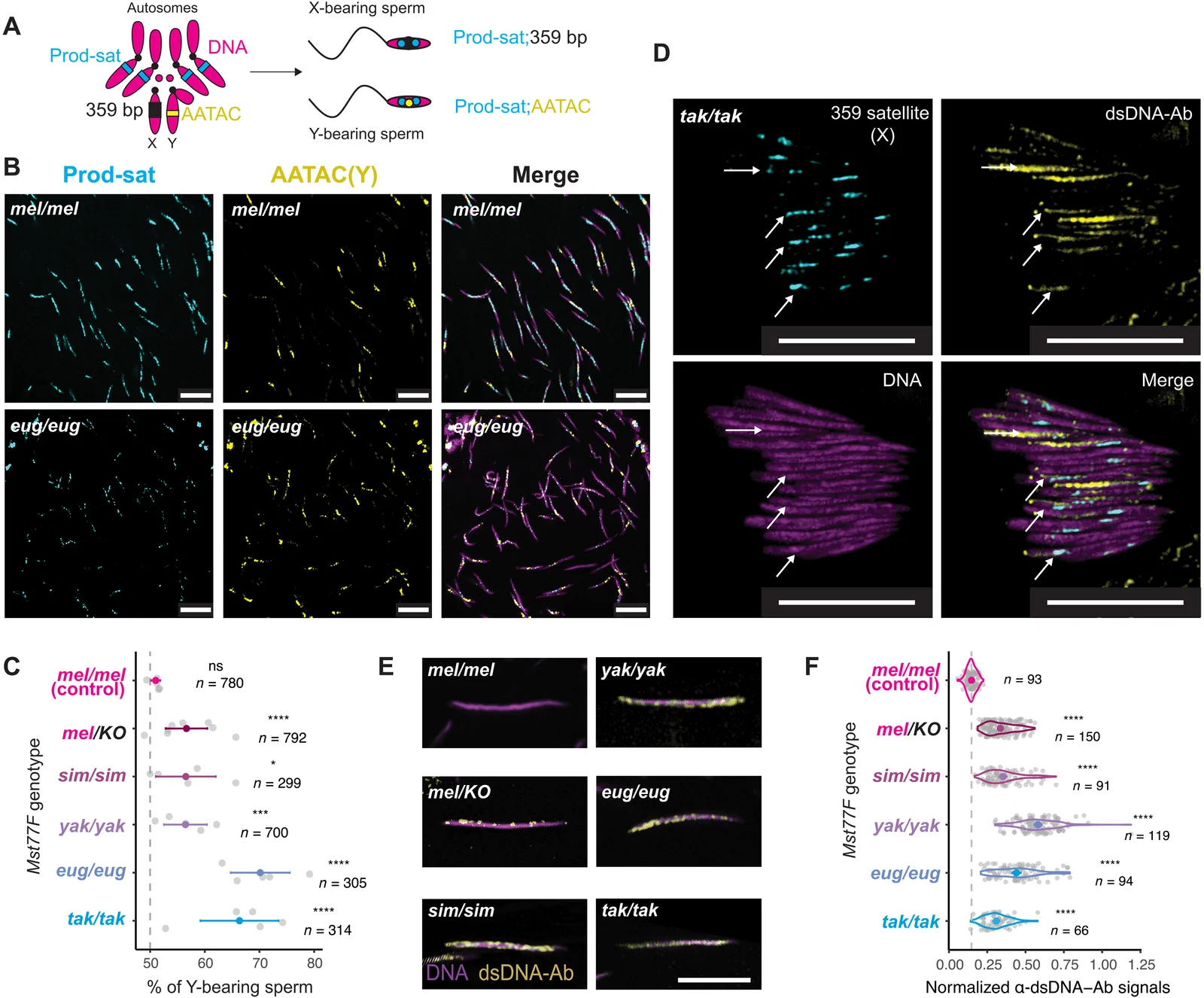
*Mst77F* gene replacements disrupt sperm chromatin condensation and decrease the proportion of X-bearing sperm. (**A**) Schematic of DNA FISH probes used to distinguish X- and Y-bearing sperm based on the Y-linked AATAC (yellow), X-linked 359-bp satellite (black), and autosomal Prod-sat (cyan) satellite repeats. DNA was stained with Hoechst 33342 (magenta). (**B**) FISH was performed using the AATAC and Prod-sat probes on seminal vesicles from homozygous *D. melanogaster* males encoding either two copies of *Mst77F-mel* or *Mst77F-eug*. The results from other genotypes are shown in fig. S6A. (**C**) Quantification of the proportion of Y-bearing sperm among total mature sperm in each of the six transgenic *Mst77F* gene replacement genotypes from (B) and fig. S6A. Each dot represents one seminal vesicle, and the dashed line represents the expected 50:50 ratio (*n* = total number of surveyed sperm in each genotype; χ^2^ test against a 50:50 ratio). (**D**) Immunostaining using an α-dsDNA antibody (yellow) was combined with FISH analyses using a probe against 359-bp satellite repeats on late-stage spermatids from *Mst77F*-tak replacement males. DNA was stained with Hoechst 33342. White arrows highlight spermatids with signals from both the X-linked 359 probe and α-dsDNA antibody. The results from other genotypes are shown in fig. S6B. (**E**) Mature sperm were stained with an α-dsDNA antibody (yellow) to assess their degree of chromatin compaction. Representative images of single sperm are shown here; seminal vesicle images are shown in fig. S6C. (**F**) α-DsDNA antibody staining was carried out on sperm from the various *Mst77F* replacement *D. melanogaster* males. Images were quantified and normalized to the Hoechst 33342 signal. (minimum 60 nuclei per genotype; unpaired Student’s *t* test; ***P < 0.001, and ****P < 0.0001). Scale bar, 10 μm for all images. Ab, antibody.

Since *Mst77F* is critical for DNA condensation, we hypothesized that delays or defects in DNA condensation might underlie reduced fertility and sex ratio distortion in males with improper *Mst77F* dosage or sequence. Under this hypothesis, X-bearing spermatids are more prone to DNA condensation defects than Y-bearing spermatids. To test this possibility, we focused on *Mst77F-tak* homozygous *D. melanogaster* flies, which have the most severe male bias in their progeny (Fig. 2B). In *Drosophila* species, X- and Y-bearing sperm resulting from a single meiotic event are produced simultaneously and undergo synchronous replication, resulting in a cyst containing 32 X-bearing and 32 Y-bearing spermatids. We performed immunostaining of spermatid cysts from *Mst77F* replacement homozygous *D. melanogaster* flies using an antibody to double-stranded DNA (α-dsDNA), which identifies decondensed spermatids (*39*). Under normal circumstances, chromatin in late spermatids and mature sperm is so tightly compacted that it completely excludes the α-dsDNA antibody. However, DNA condensation defects result in staining with the α-dsDNA antibody (Fig. 4, D to F, and fig. S6, B and C). To distinguish whether spermatids containing X or Y chromosomes might be more prone to condensation defects, we combined immunostaining using the α-dsDNA antibody with FISH using an X chromosome–specific 359-bp probe (Fig. 4A) in *Mst77F-tak* homozygous *D. melanogaster* flies. We found that, before spermatid individualization, ∼84% of decondensed spermatids are X-bearing (*n* = 57; *P* < 0.001; Fig. 4D). In some cysts, well-condensed Y-bearing spermatids appear to individualize earlier than neighboring X-bearing spermatids, which remain poorly condensed. Thus, in *Mst77F-tak* homozygous males, decondensing spermatids are predominantly X-bearing. We infer that this is also the case for the other *Mst77F* ortholog replacement males based on their mature sperm ratios (Fig. 4, B and C) and progeny sex ratios (Fig. 2).

We investigated whether chromatin condensation defects persist in mature sperm, even after sperm individualization. We observed no or minimal α-dsDNA antibody staining in mature sperm from *Mst77F-mel* homozygous *D. melanogaster* males (Fig. 4, E and F, and fig. S6C). In contrast, we observed significant α-dsDNA antibody staining in mature sperm either from hemizygous *Mst77F-mel* males or from homozygous *Mst77F-sim*, *Mst77F-yak*, *Mst77F-eug*, or *Mst77F-tak* males, consistent with defective DNA condensation persisting even in mature sperm (fig. S6C).

We conclude that the inadequate dosage of conspecific *Mst77F-mel* or the inappropriate sequence of heterospecific *Mst77F* leads to defective chromatin condensation and differential loss of X-bearing sperm during sperm development in *D. melanogaster*, resulting in meiotic drive. These same defects likely also underlie the gross alterations in sperm nuclear morphology that we observed in *Mst77F-mel* hemizygous males and homozygous *Mst77F* ortholog replacement males, but not in homozygous *Mst77F-mel* males (fig. S7A).

### *D. melanogaster Mst77F* suppresses a “killer-target” meiotic drive targeting an X-chromosomal locus

We considered two possibilities to explain the X chromosome condensation defects we observed in cases of inappropriate *Mst77F* dosage and sequence. The first possibility is akin to a “toxin-antidote” system. Under this model, the Y chromosome encodes both a trans-acting “toxin” factor that induces widespread chromatin decondensation and a cis-acting protective “antidote” factor that specifically protects Y-bearing sperm from decondensation (Fig. 5A, left). Such toxin-antidote meiotic drive systems have been previously observed on several occasions across taxa (*40*–*44*). Under this model, Y-bearing sperm would sequester both the toxin and the antidote, whereas X-bearing sperm would only sequester the toxin. Under this model, two copies of *Mst77F-mel* in wild-type *D. melanogaster* males produce sufficient Mst77F-mel to counteract the toxin, leading to equal success of X- and Y-bearing sperm (Fig. 5B, left). However, *D. melanogaster* males, which are either hemizygous for *Mst77F-mel* or homozygous for *Mst77F* orthologs, cannot sufficiently counteract the toxin, leading to impairment of X-bearing sperm and male-biased progeny (Fig. 5C, left).

**Fig. 5.**
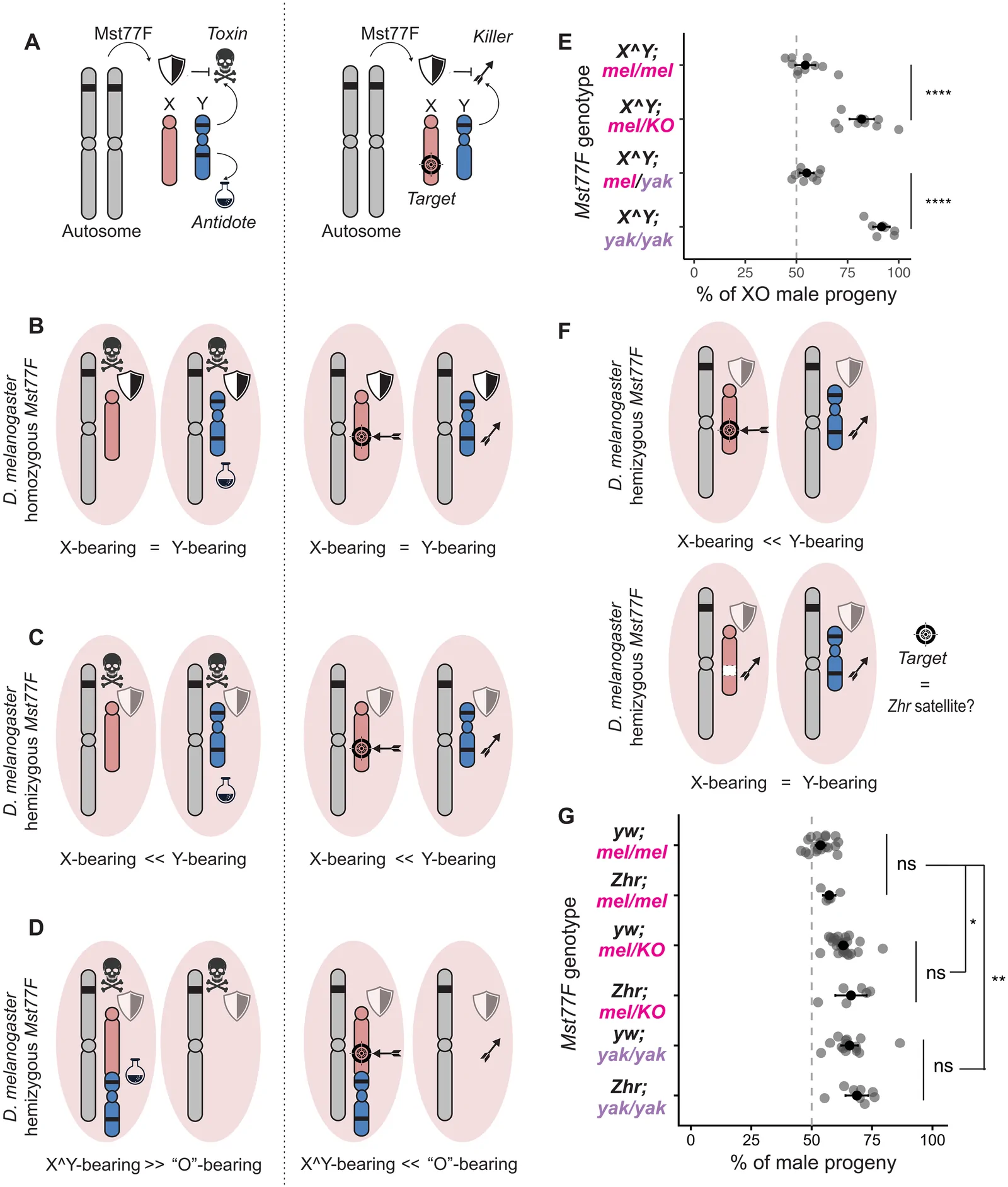
Genetic models of *Mst77F*-mediated sex ratio distortion. (**A**) Alternative mechanistic models for the Mst77F-mediated meiotic drive. We propose that autosome-encoded Mst77F suppresses a trans-acting Y-linked toxin or killer which induces meiotic drive. In the toxin-antidote model (left), the Y chromosome, but not the X chromosome, encodes another cis-acting antidote to neutralize this trans-acting Y-linked toxin. In the killer-target model (right), the X chromosome carries a target that is susceptible to this Y-linked killer. When Mst77F functions properly, it suppresses the Y-linked toxin or killer, allowing sperm bearing either the X chromosome or Y chromosome to survive symmetrically (**B**). When Mst77F function is disrupted—either by reducing gene dose (hemizygosity) or replacement by its ortholog—the activated Y-linked toxin or killer will eliminate sperm without the Y chromosome (toxin-antidote model) or carrying the X chromosome (killer-target model). In the case of males carrying independent X and Y chromosomes, the outcomes of both models are the same, leading to the overrepresentation of Y-bearing sperm (**C**). To distinguish these two models, we disrupt *Mst77F* function in males carrying the fused X^Y chromosome, which produce sperm with either an X^Y chromosome or no sex chromosome (“O”-bearing). Under the toxin-antidote model, O-bearing sperm lack the Y-linked antidote and are eliminated. Under the killer-target model, the X^Y chromosome still retains the X-linked target and is eliminated (**D**). (**E**) Dot plot illustrating the percentage of XO male progeny generated from O-bearing sperm across different Mst77F genetic backgrounds. Dashed line denotes the standard Mendelian expectation (50%). (**F**) We tested whether the X-chromosomal *Zhr* satellite represents the target of drive unleashed in hemizygous *Mst77F-mel* males. If this were the case, we expected to observe skewed sex-ratios, but only if the X-chromosome encoded the *Zhr* satellite array. (**G**) Contrary to the model in (F), we found no evidence for lower drive in males lacking the *Zhr* satellite on their X chromosome, ruling out this satellite array as the target of drive. Statistical comparisons of flies were carried out to flies encoding either homozygous *Mst77F- mel* (mel/mel) or one copy of *Mst77F- mel* and *Mst77F-yak* (mel/yak) using unpaired Student’s *t* tests (*P < 0.05, **P < 0.01, and ****P < 0.0001).

An alternative possibility is a “killer-target” system, in which a trans-acting “killer” factor acts on specific “target” chromosomal regions to induce chromosome-specific decondensation (Fig. 5A, right). Such a killer-target system is observed in sperm from *D. melanogaster* males that encode the *SD* autosomal meiotic driver, which is known to selectively kill sperm encoding large arrays of the *Responder* target satellite DNA on the second chromosome (*39*). Under this model, since the target of the drive is on the X chromosome, only X-bearing sperm will incur the consequences of the drive. Under this second model, two copies of *Mst77F-mel* produce sufficient Mst77F-mel to counteract the killer in wild-type *D. melanogaster* males, leading to equal success of X- and Y-bearing sperm (Fig. 5B, right). However, *D. melanogaster* males, which are either hemizygous for *Mst77F-mel* or homozygous for *Mst77F* orthologs, cannot sufficiently counteract the killer, leading to impairment of X-bearing sperm and male-biased progeny (Fig. 5C, right).

To distinguish between these two models, we used an attached XY (X^Y) chromosome, in which an X chromosome is fused to a Y chromosome, forming a single chromosome. X^Y males produce X^Y-bearing sperm and nullo-X sperm (lacking any sex chromosome) in equal proportions in the absence of any distortion. However, if distortion were to occur due to a toxin-antidote system, in which the Y chromosome encodes a cis-acting antidote, sperm carrying the X^Y chromosome would undergo normal sperm maturation despite the presence of X-linked sequences (Fig. 5D, left), whereas nullo-X sperm would sequester only the toxin and be impaired in chromatin condensation. This would lead to an overrepresentation of XX^Y females over XO males in the resulting progeny. Conversely, if distortion occurs due to a killer-target system that targets a locus on the X chromosome, X^Y-bearing sperm would bear the brunt of the decondensation and spermatogenesis defects, regardless of the attached Y-linked sequences (Fig. 5D, right), whereas nullo-X sperm would be unimpaired. This would lead to an excess of XO males over XX^Y females in the resulting progeny. Thus, the ratio of XX^Y females to XO males in the progeny of *Mst77F*-swap males can effectively discriminate between the two potential modes of distortion.

As a control, we first crossed wild-type *Mst77F-mel* X^Y males to females from the Oregon-R strain of *D. melanogaster*. Because of a lack of distortion, we observed a balanced 1:1 ratio of XX^Y female to XO male offspring, as expected (Fig. 5E). However, in crosses involving either hemizygous *Mst77F-mel* X^Y males or homozygous *Mst77F-yak* X^Y males, we observed a strongly distorted sex ratio of resulting progeny (>80% XO males) (Fig. 5E and data S2). These findings rule out the toxin-antidote model and strongly favor the killer-target model of distortion. They implicate a cis-acting sequence on the X chromosome as the target of distortion, leading to chromatin condensation failure and sperm maturation defects observed in *Mst77F*-swap males.

Given the gross condensation defects, we hypothesized that this cis-target sequence must be abundant and either unique to or substantially enriched on the *D. melanogaster* X chromosome. On the basis of these dual criteria, we investigated whether the X-linked target is the 1.688 satellite (also known as the 359-bp satellite or *Zhr*, for *Zygotic hybrid rescue*), a multimegabase pericentromeric satellite DNA that recently expanded on the X chromosome of *D. melanogaster* (*45*). If *Zhr* represents the X-linked target of distortion, then we predicted that an X chromosome carrying a large *Zhr* pericentromeric deletion (Δ*Zhr*) would be less susceptible to sex ratio distortion caused by *Mst77F* mismatches (Fig. 5F). Contrary to this prediction, we found that the Δ*Zhr* X chromosome was not significantly less sensitive to sex ratio distortion seen in wild-type *Mst77F-yak* homozygous *D. melanogaster* males (Fig. 5G). Although our findings rule out *Zhr* as the target of drive, they indicate that an as-yet-unknown *D. melanogaster* X chromosomal locus is the target of the sex chromosome meiotic drive unleashed in *Mst77F*-swap males.

### *D. yakuba Mst77F* knockouts are fertile but undergo sex ratio distortion

The crucial function of *Mst77F* in male fertility is unlikely to be universally conserved across *Drosophila* species, given its recent emergence ∼20 Ma ago and its loss in at least one lineage (the *montium* group). To clarify whether fertility, suppression of meiotic drive, or both are the primary selective pressures driving the rapid evolution of *Mst77F*, we generated a CRISPR-Cas9–mediated knockout of *Mst77F* in *D. yakuba*, a closely related species that diverged from *D. melanogaster* only 6 Ma ago (fig. S8) (*35*, *46*). We found that, unlike in *D. melanogaster*, *Mst77F-yak* knockouts in *D. yakuba* exhibited reduced fertility but are not completely sterile (Fig. 6A), suggesting that *Mst77F*’s essential role in male fertility is likely a recently derived function in *D. melanogaster.*

**Fig. 6.**
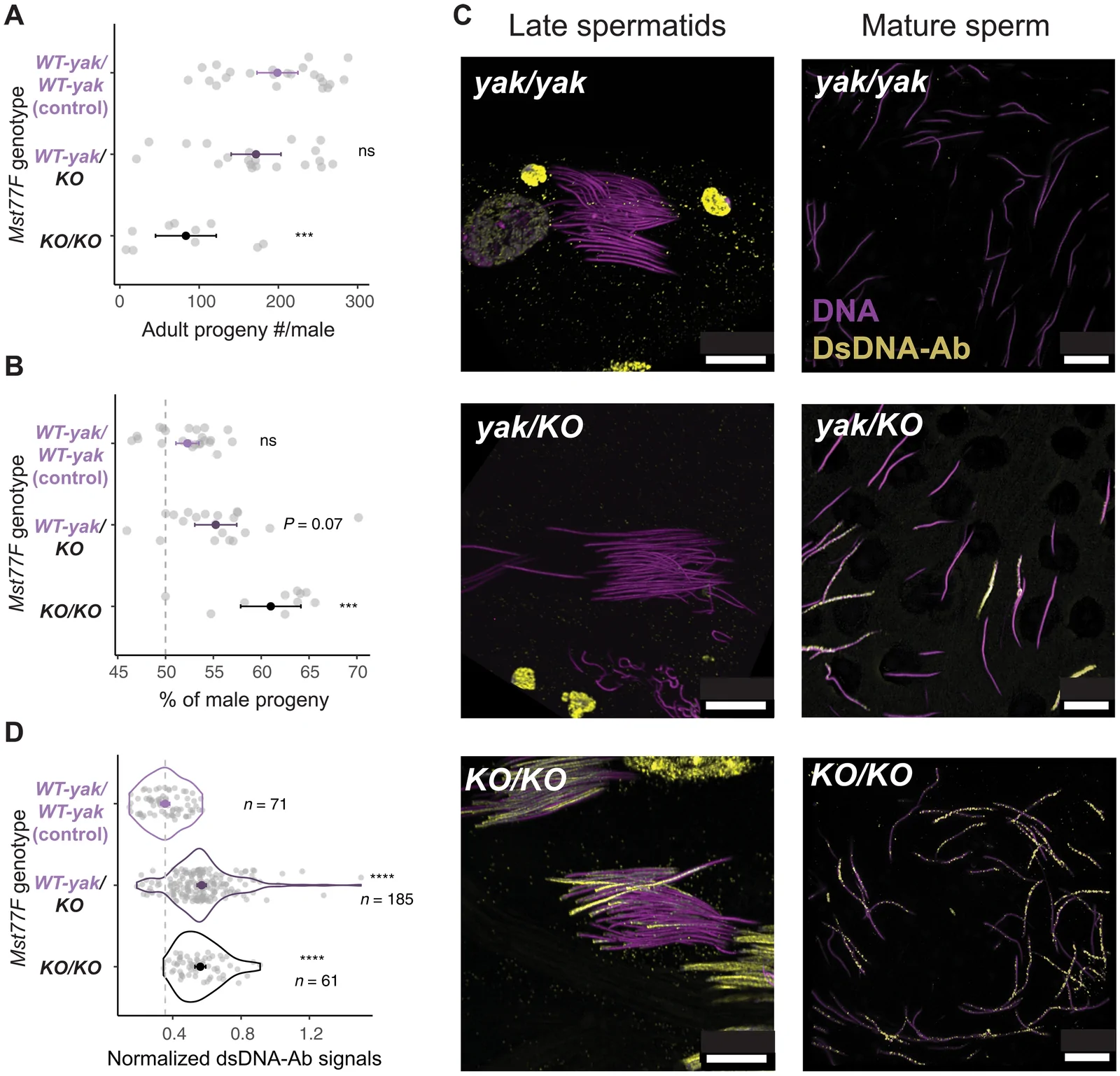
*Mst77F* knockouts in *D. yakuba* are fertile but have sex ratio distortion. (**A**) Fertility of *D. yakuba* males carrying no, one, or two copies of wild-type *Mst77F-yak*. (**B**) Adult progeny sex ratios from *D. yakuba* males carrying no, one, or two copies of *Mst77F-yak.* Deviation from a 50:50 Mendelian expectation was measured using a χ^2^ test. (**C**) Late spermatids and mature sperm from different *D. yakuba* males were stained with an α-dsDNA antibody (yellow) to assess their degree of chromatin compaction. Scale bars, 10 μm for all images. (**D**) Quantification of α-dsDNA antibody staining in sperm from wild-type, hemizygous, and *Mst77F-yak* knockout *D. yakuba* males. Statistical analyses were carried out using unpaired Student’s *t* tests (***P < 0.001, and ****P < 0.0001).

We found that *D. yakuba* males lacking one or both copies of *Mst77F-yak* produced male-biased offspring in a dose-dependent manner: Homozygous knockout males show a more pronounced bias than hemizygous males (Fig. 6B). Consistent with our findings in *D. melanogaster*, we found that the depletion or absence of *Mst77F-yak* in *D. yakuba* males led to sperm DNA compaction defects (Fig. 6, C and D) and abnormal nuclear size in mature sperm (fig. S7B). While these compaction defects were pervasive, a subset of mature sperm remained viable and competent for fertilization, mirroring the incomplete penetrance of condensation failure observed in a particular *SD* background (*47*).

These phenotypes of *D. yakuba*, including the male-biased offspring, are reminiscent of those observed in crosses involving either hemizygous *Mst77F-mel* or homozygous *Mst77F-yak D. melanogaster* males, both of which are fertile yet exhibit sex ratio distortion (Fig. 2). These findings suggest that both endogenous copies of *Mst77F-yak* are required to suppress delays or defects in sperm DNA compaction that would otherwise distort the progeny sex ratio in *D. yakuba*. Given the resulting male-biased progeny, we infer that these chromatin condensation defects also preferentially affect X–bearing spermatids and mature sperm, as in *D. melanogaster*. On the basis of our detailed investigation of *Mst77F* in both *D. melanogaster* and *D. yakuba* males, we conclude that its rapid evolution across *Drosophila* species is more likely to be driven by its previously undiscovered role in suppressing meiotic drive than by its previously described essential function in male fertility.

## DISCUSSION

Novel biological pressures shaping cellular processes can lead to unexpected signatures of genetic innovation, even in genes critical for viability or fertility (*48*). Despite their essential roles in male fertility in mammals and *Drosophila* species (*2*), protamine genes are among the most rapidly evolving protein-coding genes in many animals. Yet, the underlying causes of protamine evolution have remained unclear. Here, using genetic and cytological tools available in *D. melanogaster* and related species, we performed in vivo gene replacements to show that the rapid evolution of *Mst77F* protamine, which is essential for male fertility in *D. melanogaster*, ensures proper DNA condensation in sperm carrying either X or Y chromosomes during spermatogenesis. Failure to do so results in decreased fertility and sex ratio distortion. Our findings indicate that suppression of competition between X and Y chromosomes may be a primary driver of the rapid evolution of protamines.

Our findings indicate that the *Mst77F* protamine, which plays a crucial role in sperm DNA compaction, may suppress meiotic drive by ensuring balanced representation of X and Y chromosomes in mature sperm. Disruptions in *Mst77F* dosage or sequence predominantly affect X chromosomes carrying specific targets, enabling Y-bearing sperm to outcompete X-bearing sperm and skew sex ratios. In contrast, a complete loss of *Mst77F* impairs condensation of all chromosomes, leading to complete male sterility in *D. melanogaster*. *Mst77F* might compact and directly protect an X-linked target from the drive. Y-linked duplications and expansions of protamines, including *Mst77Y* in *D. melanogaster* (*2*) or *Mst33A* in *D. yakuba* (*2*), may interfere with *Mst77F* function, favoring their transmission by disrupting X-bearing sperm and prompting rapid evolutionary adaptation of *Mst77F* to evade such antagonism and maintain sex ratio parity. Alternatively, *Mst77F* might suppress sex chromosome–linked sperm killers, which are selfish elements that bias their own transmission by disabling sperm carrying the opposing sex chromosome.

Such drivers need not reside on the Y. Our previous phylogenomic survey suggested that protamine-derived killers can arise on either the X or the Y, with the homologous sex chromosome serving as the target in each case (*2*). In this view, an autosomal protamine such as *Mst77F* is well positioned to act as a neutral arbiter, suppressing whichever sex chromosome gains a transmission advantage and thereby preserving the Fisherian 50:50 sex ratio that benefits the rest of the genome (*30*, *31*). Loss of *Mst77F* could unleash these selfish elements, distort sex ratios, and cause sterility when genome-wide condensation fails. Thus, the widespread and rapid evolution of *Mst77F* and other protamines might reflect an ongoing evolutionary arms race with such sex-linked drivers. Future studies investigating the relationship between protamine evolution and the presence of sperm killers may illuminate the mechanisms underlying selfish genetic elements and their effects on male reproductive success and sperm chromatin integrity.

Our results, together with the genomic distribution of protamine genes, suggest that distinct protamines may have evolved to counter distinct meiotic drivers. *Mst77F* suppresses a sex chromosome drive system whose target is X-linked, whereas the autosomal protamines *ProtA* and *ProtB* are implicated in suppressing the autosomal driver *SD* (*38*) but do not affect sex chromosome meiotic drive. Because the transmission conflict that fuels drive is most acute between the sex chromosomes, we expect sex chromosome drive, which targets either the X or the Y in different lineages, to be a particularly recurrent pressure shaping protamine evolution (*2*).

Our findings reveal that both *Mst77F-mel* and *Mst77F-yak* suppress sex chromosome meiotic drive in their respective species. In contrast to their roles in male fertility, both genes are haploinsufficient to suppress sex ratio distortion, even in the presence of other protamines. Moreover, loss of competition between X- and Y-bearing sperm due to a rare evolutionary X^Y fusion in the *montium* group of *Drosophila* species correlates with the loss of *Mst77F* and other protamines (*2*). These findings suggest that the retention, dosage, and rapid evolution of *Mst77F* and likely other protamine genes are associated with their role in suppressing meiotic drive during spermatogenesis. Our work highlights the power of an in vivo gene replacement strategy to investigate long-standing conundrums about the biological causes and consequences of the rapid evolution of genes essential for fertility and viability. It also highlights that, despite its ubiquity, Mendelian inheritance is ultimately a highly fragile truce.

## MATERIALS AND METHODS

### CRISPR-Cas9 knockout of *Mst77F* in *D. melanogaster* and *D. yakuba*

To generate the *Mst77F* knockout strain, we adapted the *pCFD4-U6:1_U6:3* tandem gRNAs plasmid (Addgene #49411) with two gRNAs targeting either the 5′ or 3′ end of *Mst77F*. For the repair construct, we amplified four fragments via polymerase chain reaction (PCR): the 3×P3 *DsRed* marker, the backbone of pDsRed-attP (Addgene #51019), and ∼1 kb of homologous sequences flanking the upstream and downstream regions of *Mst77F*. These four fragments were then annealed using NEBuilder HiFi DNA Assembly Master Mix into one “repair construct.” We used the Q5 Site-Directed Mutagenesis Kit (NEB, catalog no. E0554S) to mutate the gRNA target PAM sites on the repair construct to avoid recutting of the repair construct by CRISPR-Cas9 and the gRNAs.

For *D. melanogaster*, these two constructs (one encoding the repair construct and the other encoding the gRNAs) were coinjected into the *y[1] M{GFP[E.3*×*P3]=vas-Cas9.DsRed-}ZH-2A w[1118]* strain (BDSC stock #55821) embryos by BestGene Inc. The transgenic flies were backcrossed to the *yw* strain, selected using the DsRed marker, and then balanced using the *TM3*, *Sb* balancer chromosome. We used PCR to validate the successful *Mst77F-mel* knockout (fig. S1B). The *Mst77F-mel* knockout was further confirmed using complementation tests with the *Mst77FΔ1* knockout flies (*34*) (gift from B. Loppin) (fig. S1B).

For *D. yakuba*, we similarly constructed gRNA and repair template plasmids as described above. These two constructs were coinjected into a *D. yakuba* strain encoding *nanos-cas9* on the X chromosome (*49*) (gift from D. Stern) by Genetivision Inc. The transgenic flies were backcrossed to a *w* strain of *D. yakuba* and selected using the *DsRed* marker (fig. S9A). We used PCR to validate the successful *Mst77F-yak* knockout (fig. S9B). The *D. yakuba* stocks are maintained as heterozygotes by selecting the *DsRed* marker every two to three generations. All primers are listed in data S1.

### Recombinase-mediated in vivo gene replacements at the endogenous *Mst77F* locus in *D. melanogaster*

For the gene replacement and rescue experiments in *D. melanogaster*, we used the attP site in the integrated repair construct of *Mst77F KO* flies. First, we removed the *DsRed* marker in *Mst77F KO* flies by crossing the knockout strain with *Cre* recombinase–carrying males (BDSC #1501). This removes the *DsRed* gene, which is flanked by *loxP* sites. The resulting *Mst77F KO/TM6B*, *Cre* males were backcrossed to the maternal *Mst77F KO/TM3*, *Sb* strain. The resulting *DsRed*-negative flies carrying the *TM3*, *Sb* balancer were selected to establish a stable stock.

Next, we amplified the coding sequences of different *Mst77F* orthologs from various species using reverse transcription PCR (RT-PCR) from male RNA. We cloned these *Mst77F* copies flanked by the promoter and untranslated regions from *D. melanogaster*, into a vector carrying attB and 3×P3 *DsRed* (gift from C. Schroeder) using NEBuilder HiFi DNA Assembly Master Mix.

To generate the overexpression construct, the PiggyBAC backbone (gift from C. Schroeder), containing green fluorescent protein and mini-white markers, was linearized via double digestion with SbfI-HF and NotI-HF. Subsequently, the *Mst77F-yak* transgene flanked by *D. melanogaster* promoter and untranslated region sequences was subcloned from the attB donor vector into the linearized PiggyBAC vector using NEBuilder HiFi DNA Assembly Master Mix.

These constructs were coinjected along with a helper plasmid encoding either ΦC31 integrase or *piggyBac* transposase into *Mst77F-mel KO [DsRed-flox]/TM3*, *Sb* flies by BestGene Inc. The resulting transgenic flies were backcrossed with *Mst77F-mel KO [RFP-flox]/TM3*, *Sb* to ensure an identical genetic background. The resulting *DsRed*-positive flies were selected and maintained using the *TM3*, *Sb* balancer chromosome. All primers are listed in data S1.

### Constructing chimeras between *Mst77F-mel and Mst77F-yak*

We used PCR to amplify different regions of *Mst77F-mel* and *Mst77F-yak* using the plasmids created in the previous section. We then constructed attB clones with the *Mst77F-mel/Mst77F-yak* chimeras by annealing the fragments using NEBuilder HiFi DNA Assembly Master. In this way, we generated vectors carrying attB, 3×P3 DsRed, and different *Mst77F-mel/Mst77F-yak* chimeras. These constructs were again injected into *Mst77F-mel KO [DsRed-flox]/TM3*, *Sb* flies by BestGene. The resulting transgenic flies were backcrossed with *Mst77F-mel KO [DsRed-flox]/TM3*, *Sb* to ensure an identical genetic background. *DsRed*-positive flies were selected and maintained using *TM3, Sb* balancer chromosomes. All primers are listed in fata S1.

### Sequencing and RT-PCR

We extracted genomic DNA from individual flies by grinding a single fly in a buffer containing 10 mM tris-HCl (pH 8), 1 mM EDTA, 25 mM NaCl, and proteinase K (200 μg/ml). The mixture was incubated at 37°C for 30 min, followed by heat inactivation of proteinase K at 95°C for 3 min. For RNA extraction, we homogenized flies or specific tissues in TRIzol reagent (Invitrogen), added chloroform, and followed the manufacturer’s protocol for RNA extraction using the RNA Clean and Concentrator kit (Zymo Research) with deoxyribonuclease I (DNase I) to purify the RNA. Samples were treated with DNase I (Zymo Research) and further purified and concentrated using the same kit if there was still DNA contamination. The resulting RNA was reverse-transcribed into cDNA using SuperScript III First-Strand Synthesis (Invitrogen Inc.). All primers are listed in data S1.

### Cross schemes and fertility assays

To visualize histone removal, we introduced *His2AV-mRFP* and *cid-GFP* from BDSC strain #91708 into *Mst77F* replacement backgrounds. Females from the reporter strain were crossed to males carrying either the *Mst77F-mel* or *Mst77F-yak* transgene. Resulting virgin females (*His2AV-mRFP*, *cid-GFP/Mst77F*) were backcrossed to the paternal transgenic lines. Last, progeny carrying both fluorescent reporters and the transgenic *Mst77F* allele balanced over *TM3*, *Sb* were selected to establish stable stocks. A similar cross was used to increase the copy number of *Mst77F-yak* on the third chromosome.

To introduce attached X^Y chromosomes into *Mst77F*-deficient backgrounds, we used a strain carrying both attached X^X and attached X^Y chromosomes (BDSC strain #9460). We first crossed males from our *Mst77F-KO* or *Mst77F-yak* strain to females from #9460. The resulting X^X/Y females were then backcrossed to #9460 males to get X^Y/Y males carrying the *Mst77F-KO* or *Mst77F-yak*, identified by their respective DsRed selection markers. These X^Y/Y males were backcrossed once more to #9460 females again to segregate out the extra Y chromosome, yielding X^Y males carrying the desired *Mst77F* allele. We maintain these experimental stocks by selecting for the DsRed marker in each generation.

To test whether the *Zhr* locus (comprising the 359-bp satellite repeats) is the target locus of *Mst77F*-regulated meiotic drive, we genetically removed the *Zhr* region from the *Mst77F* transgenic background. First, the X chromosome from the strain (BDSC strain #25140) with a deletion of *Zhr* (Δ*Zhr*) was introduced to the TM3/TM6B double-balancer background to generate a stable base stock, Δ*Zhr*; TM3/TM6B. To introduce the specific Mst77F variants, Δ*Zhr*; TM3/TM6 females were crossed to males from the *Mst77F-KO/*TM3, *Mst77F-mel/*TM3, or *Mst77F-yak/*TM3 stock. The resulting Δ*Zhr; Mst77F-KO/*TM3, Δ*Zhr;Mst77F-mel/*TM3, or Δ*Zhr; Mst77F-yak/*TM3 males were backcrossed to the baseline to establish balanced stocks (Δ*Zhr; Mst77F-KO/*TM3, Δ*Zhr; Mst77F-mel/*TM3, and Δ*Zhr; Mst77F-yak/*TM3). To test a potential dosage effect, we crossed the Δ*Zhr*; *Mst77F-mel* strain and the Δ*Zhr*; *Mst77F-KO* strain to yield trans-heterozygous experimental males (Δ*Zhr*; *Mst77F-mel/Mst77F-KO*). Last, these males were crossed to *yw* or OreR females at a 1:5 ratio for 9 days in total, and their female and male progeny were scored.

To measure male fertility and progeny sex ratios, we crossed each 2- to 6-day-old tester male to five 2- to 6-day-old virgin females from the wild-type Oregon-R *D. melanogaster* strain, an attached X^X strain (BDSC strain #9460), or a *white D. yakuba* strain (gift from D. Stern) at 25° or 29°C. For *D. melanogaster*, we transferred the mating pairs to new vials every 3 days and counted all resulting offspring from the first 9 days. For *D. yakuba*, we transferred the mating pairs to new vials every 7 days and counted all resulting offspring from the first 21 days. All raw results are listed in data S2.

### Mst77F dephosphorylation assays and Western blot

To extract Mst77F, testes from 2-day-old virgin males were dissected in the chilled dissection buffer (1× Cutsmart buffer, 150 mM NaCl, and 1× protease inhibitor). Samples were then lysed in the dissection buffer with 0.1% Triton X-100 through five freeze-thaw cycles using liquid N_2_, followed by centrifugation at 15,000*g* for 10 min at 4°C. The resulting supernatant was divided into equal aliquots and treated with either 5 U of Quick-CIP (NEB) or an equivalent volume of lysis buffer (control). Following a 1-hour incubation at 37°C with 700 rpm shaking, samples were mixed with 4× Laemmli buffer and heated at 90°C for 5 min.

Electrophoresis was performed using 12% SDS–polyacrylamide gel electrophoresis at 100 V, followed by a 1-hour transfer to nitrocellulose membranes at 100 V. After blocking with Hyblock buffer for 5 min, the membrane was incubated overnight at 4°C with primary antibodies: mouse anti-FLAG (1:2000; Sigma-Aldrich, F3165) and rabbit anti–α-tubulin (1:5000; Abcam, ab52866) diluted in 5% bovine serum albumin (BSA) in TBST (0.05% Tween 20). Following three washes with TBST, the membrane was incubated with fluorescent secondary antibodies—goat α-mouse (1:10,000; LI-COR, 926-32210) and goat α-rabbit (1:10,000; Abcam, ab175773)—in 5% BSA in TBST for 1 hour at room temperature. After three final washes in TBST, the blots were visualized using an Invitrogen iBright FL1500 Imaging System. All raw results are listed in data S3.

### Quantification of seminal vesicle sizes

Testes with seminal vesicles were carefully dissected from 5-day-old virgin males in 1× phosphate-buffered saline (PBS) buffer and mounted in 30 μl of 1× PBS buffer to avoid squashing the tissue. Images were captured under bright-field illumination (ZEISS Scope A1), and the total two-dimensional surface area (in square micrometers) was manually outlined and calculated via ZEISS Zen 3.9. All raw results are listed in data S4.

### Immunofluorescence assays

For immunofluorescence, testes from 1- to 3-day-old virgin male flies were dissected and fixed in 4% paraformaldehyde (PFA) for 20 min at room temperature. The samples were permeabilized in 0.3% sodium deoxycholate in PBS with 0.1% Triton X-100 (PBST) for 15 min, repeated twice, followed by three washes with PBST. Testes were then blocked for 1 hour in 3% BSA in PBST (blocking buffer) and incubated overnight with primary antibodies (1:200 rabbit α-FLAG; Proteintech, 0543-1-A and 1:1000 mouse α-DsDNA; Abcam, 27156) diluted in the blocking buffer at 4°C. On the second day, the testes were washed three times with PBST and treated with corresponding secondary antibodies (1:1000) diluted in the blocking buffer at room temperature for 2 hours, with Hoechst 33342 (1:1000; Invitrogen) added for DNA staining. After three additional washes with PBST and a final wash with PBS, the testes were mounted onto slides using SLOWFADE antifade mounting medium with 4′,6-diamidino-2-phenylindole (DAPI; Invitrogen Inc., S36938) for imaging.

### Fluorescence in situ hybridization assays

For FISH, testes were dissected from 1- to 3-day-old virgin male flies and fixed in 4% PFA for 30 min at room temperature. Similarly for immune-FISH, the samples from immunostaining were postfixed in 4% PFA for 6 min at room temperature. The samples were treated with ribonuclease A (Invitrogen) in PBST for 10 min. After treatment, the samples were washed twice with 2× saline sodium citrate with 0.1% Tween 20 (2× SSCT) and 1 mM EDTA, followed by a single wash with hybridization buffer. We then treated the samples with 50 μl of hybridization buffer containing 50 to 100 ng of probes (see data S1) and 1 mM EDTA and denatured the samples at 95°C for 8 min before hybridizing them overnight at 37°C in the cap of an Eppendorf tube sealed with heat sealing film. The next day, after washing three times with 4× SSCT and 0.1× SSC, the testes were mounted onto slides using SLOWFADE antifade mounting medium with DAPI (Invitrogen Inc., S36938) for imaging. The solution and details of the FISH protocol followed the procedure outlined previously (*50*), except that we supplemented the 2× SSCT with 1 mM EDTA during hybridization (*51*).

### Imaging analyses and sperm shape assays

We used a DragonFly spinning disk confocal microscope with a 100× oil-immersion objective to capture all images, using Fusion software for acquisition. For the FISH experiment, sperm were categorized on the basis of distinct probe signals. To measure sperm length and fluorescence intensity in immunostaining, we used the surface modeling function in IMARIS to identify long, straight sperm nuclei. Following an additional round of manual curation, we calculated sperm length by doubling the ellipsoid axis length C value. To quantify signal intensity, we measured the fluorescence of the α-dsDNA antibody and normalized it to the Hoechst 33342 signal to correct for signal decay across the *z* axis. All raw results are listed in data S5.

### RNA sequencing and analyses

We collected 5 to 10 pairs of testes from 2- to 3-day-old virgin male flies and carefully removed the accessory glands on ice. Testes were homogenized in 200 μl of TRIzol, followed by the addition of 40 μl of chloroform. After centrifugation at 13,000*g* for 15 min at 4°C, we collected 100 μl of the aqueous phase and purified the RNA using the Zymo RNA Clean and Concentrator kit (R1015) with on-column DNase I treatment. The RNA was dissolved in 20 μl of ddH_2_O. If NanoDrop or PCR detected DNA or phenol contamination, we repeated the purification using the same Zymo kit. Purified total RNA was submitted to Novogene for poly(A) enrichment, library preparation, and sequencing on the NovaSeq X platform, generating at least 6 Gb of 150-bp paired-end reads per sample. RNA sequence reads are in National Center for Biotechnology Information (NCBI)’s SRA under BioProject PRJNA1290820.

To quantify the expression of *Mst77F* orthologs in *Mst77F* gene replacement males, we generated a custom transcriptome reference based on FlyBase r6.63, in which the original *Mst77F-mel* transcript sequence was removed and replaced with FLAG-tagged *Mst77F-mel* and *Mst77F-yak* sequences from the constructs. We then quantified transcript abundance using SALMON v1.10.1 (*52*) using the following parameters: --validateMappings --allowDovetail --recoverOrphans --gcBias --seqBias. For comparison, RNA sequencing data from wild-type testes (*w^1118^* strain) were obtained from the publicly available dataset PRJNA687271 (*53*).
