# Supplementary material for: Rapid protamine evolution suppresses meiotic drive in Drosophila
Source: Sci Adv. 2026 Oct 7;12(41):eaeb5514. doi: 10.1126/sciadv.aeb5514 (PMC13644785; doi:10.1126/sciadv.aeb5514)
Supplement: Supplementary file 1 [file sciadv.aeb5514_sm.pdf]

Science Advances
AAAS

Supplementary Materials for

Rapid protamine evolution suppresses meiotic drive in *Drosophila*

Ching-Ho Chang *et al.*

Corresponding author: Ching-Ho Chang, chinghochang@as.edu.tw

Sci. Adv. **12**, eaeb5514 (2026)
DOI: 10.1126/sciadv.aeb5514

The PDF file includes:

Figs. S1 to S9
Legends for data S1 to S5

Other Supplementary Material for this manuscript includes the following:

Data S1 to S5

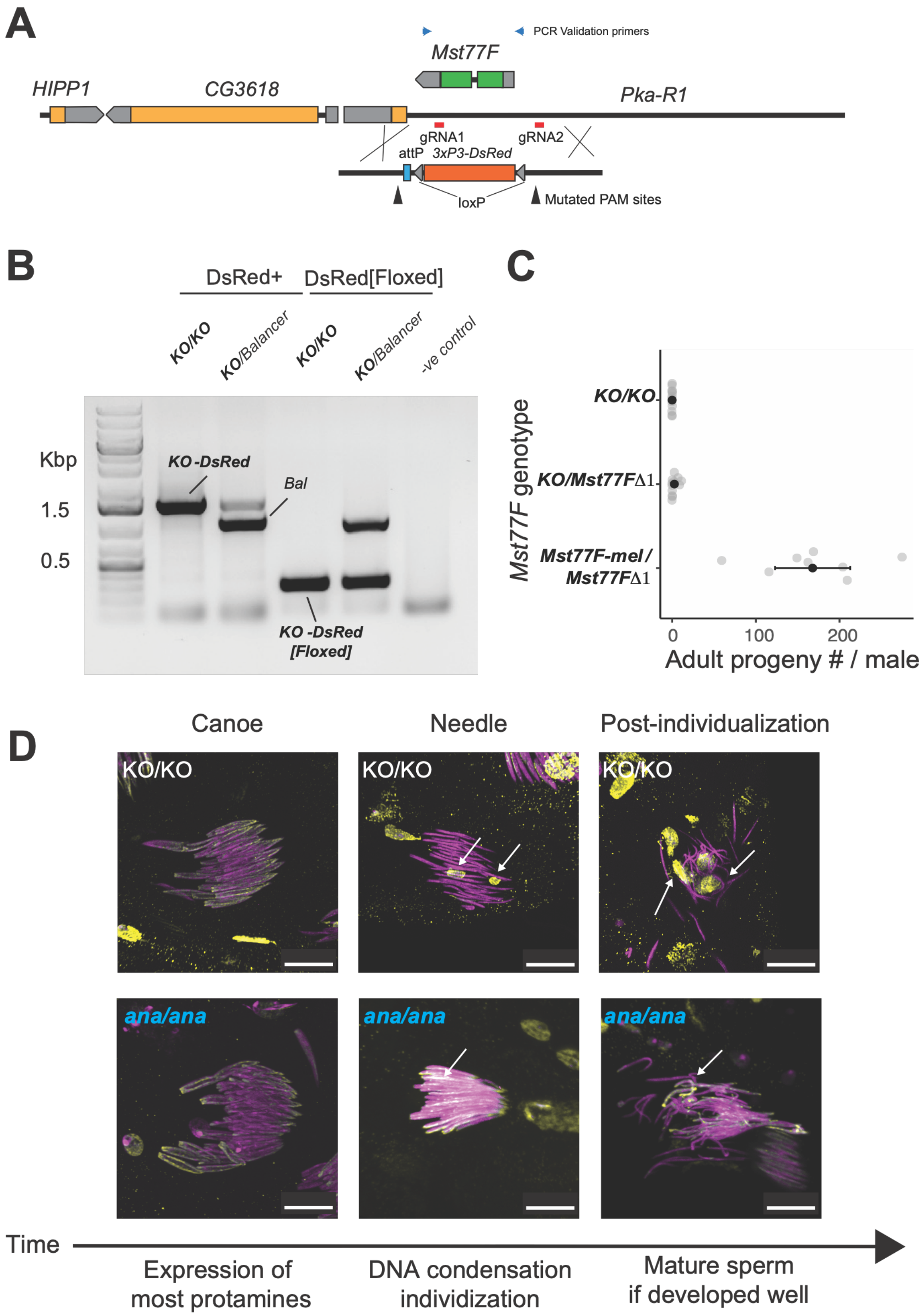
A
PCR Validation primers
Mst77F
HIPP1
CG3618
Pka-R1
gRNA1
gRNA2
attP
3xP3-DsRed
loxP
Mutated PAM sites
B
DsRed+
DsRed[Floxed]
KO/KO
KO/Balancer
KO/KO
KO/Balancer
-ve control
Kbp
1.5
0.5
KO-DsRed
Bal
KO-DsRed [Floxed]
C
Mst77F genotype
KO/KO
KO/Mst77FΔ1
Mst77F-mel / Mst77FΔ1
0
100
200
Adult progeny # / male
D
Canoe
Needle
Post-individualization
KO/KO
ana/ana
Time
Expression of most protamines
DNA condensation individization
Mature sperm if developed well

Fig. S1: *Mst77F* knockout and replacement in *Drosophila melanogaster*. (A) Schematic representation of the genomic region flanking the *Mst77F* locus in *D. melanogaster*. The positions of the flanking *HIPP1*, *CG3618*, and *Pka-R1* genes are shown. The CRISPR/Cas9 strategy for generating the knockout (*KO*) allele is depicted, including the locations of guide RNAs gRNA1 and gRNA2, and a repair construct carrying a 3×P3-DsRed marker, an attP site, and loxP sites. PAM sites were mutated in the repair construct to prevent re-cutting by CRISPR/Cas9. PCR validation primer sites are shown as blue arrowheads. **(B)** PCR genotyping of flies carrying the *KO* and floxed alleles shows bands corresponding to the *Mst77F-mel KO*-DsRed, *KO*-DsRed/balancer (*Bal*), *KO*-DsRed[floxed], *KO*-DsRed[floxed]/balancer flies, as well as a negative control. **(C)** *KO/KO* males and trans-heterozygote *KO*/*Mst77FΔ1* (an independently derived *Mst77F* knockout allele(*34*)*)* are sterile. This male sterility can be complemented by FLAG-tagged *Mst77F-mel*, indicating that *Mst77F-mel* functions as a wild-type allele. **(D)** α-DsDNA antibody staining of sperm from the *KO*/*KO D. melanogaster* males and *D. melanogaster* homologous for *Mst77F-ana*. Both males cannot produce mature sperm and shows chromatin condensation defects in sperm nuclei (which leads to α-DsDNA antibody staining) during individualization and post-individualization stages of spermatogenesis. DNA was stained with Hoechst 33342 (magenta). Scale bar = 10 µm for all images.

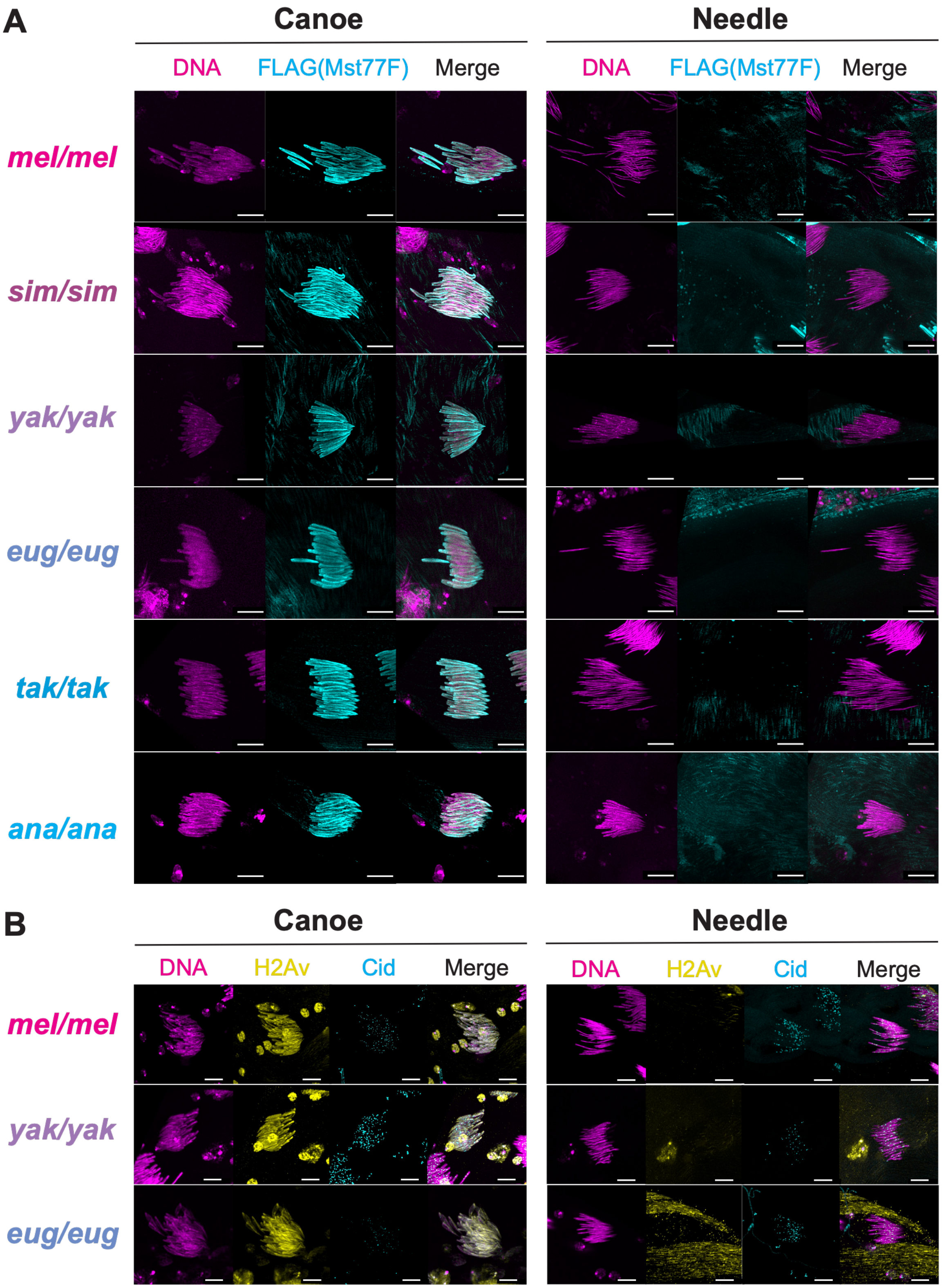
A
Canoe
Needle
DNA
FLAG(Mst77F)
Merge
mel/mel
sim/sim
yak/yak
eug/eug
tak/tak
ana/ana
B
Canoe
Needle
DNA
H2Av
Cid
Merge
mel/mel
yak/yak
eug/eug

Fig. S2: Expression and localization of *Mst77F* orthologs and histones during spermatid maturation stages. Testes from transgenic flies expressing different *Mst77F* orthologs were immunostained and imaged at the Canoe and Needle (individualization) stages of spermatogenesis. **(A)** FLAG-tagged Mst77F was detected using α-FLAG antibody (cyan), DNA was stained with Hoechst 33342 (magenta). Mst77F is known to be expressed at the canoe stage and retained in mature sperm. However, during and after the needle stage, sperm nuclei are highly compacted, preventing antibody penetration. Signal detected outside spermatid nuclei reflects nonspecific binding. **(B)** Histone 2A variant (*His2Av*) and the centromeric histone (*cid*) were visualized via endogenously tagged *His2Av-RFP* (yellow) and *cid-GFP* (cyan). Scale bar = 10 μm for all images.

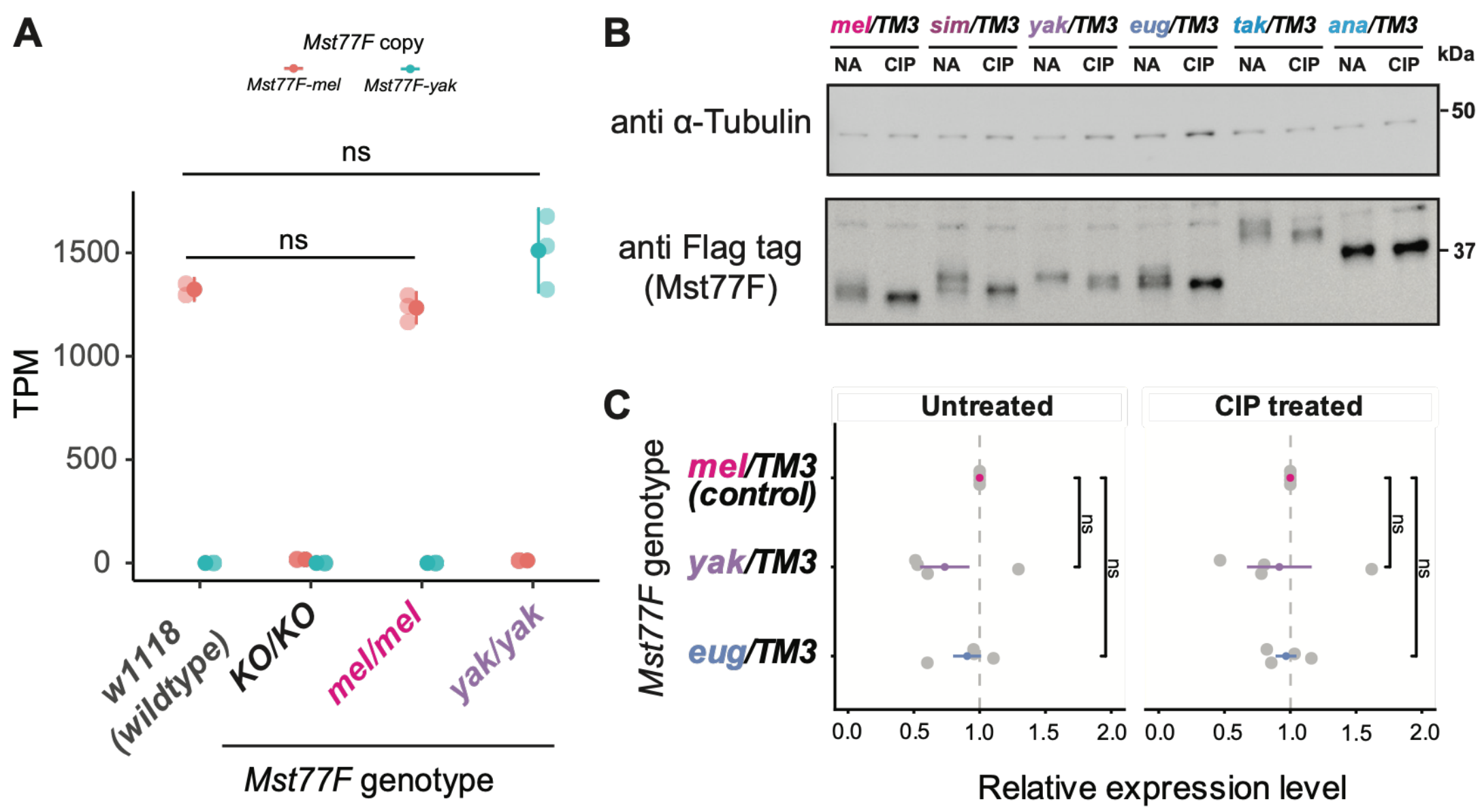

Fig. S3. Expression levels and phosphorylation of *Mst77F* transgenes in testes. (**A**) We quantified the expression of *Mst77F* in the testes of a wild-type strain (*w1118*), *an Mst77F* knockout strain (*KO/KO*), a rescued homozygous *Mst77F-mel* strain (*mel/mel*), and a homozygous *Mst77F-yak* replacement strain (*yak/yak*) using RNA-seq. To avoid mapping bias and distinguish the expression of *Mst77F-mel* and *Mst77F-yak*, we mapped the reads to a modified Flybase transcriptome reference, in which the original *Mst77F* transcript sequence was replaced with FLAG-tagged *Mst77F-mel* and *Mst77F-yak* sequences from the constructs we used. Statistical analyses were carried out using unpaired Student's *t*-tests, comparing with the wild-type flies. (**B**) Phosphorylation analysis via Western Blot of transgenic Mst77F-FLAG proteins across species. A downward shift in the anti-Flag signal following dephosphorylation treatment via Calf Intestinal Phosphatase (CIP) indicates phosphorylation of all Mst77F proteins except the most distant Mst77F-*ana*. α-tubulin serves as a loading control. (**C**) Quantification of three different Mst77F protein levels (normalized to α-tubulin) in the presence or absence of CIP treatment. Significance was determined by unpaired Student's *t*-tests (*p<0.05; **p<0.01; ***p<0.001; ****p < 0.0001; ns, not significant).

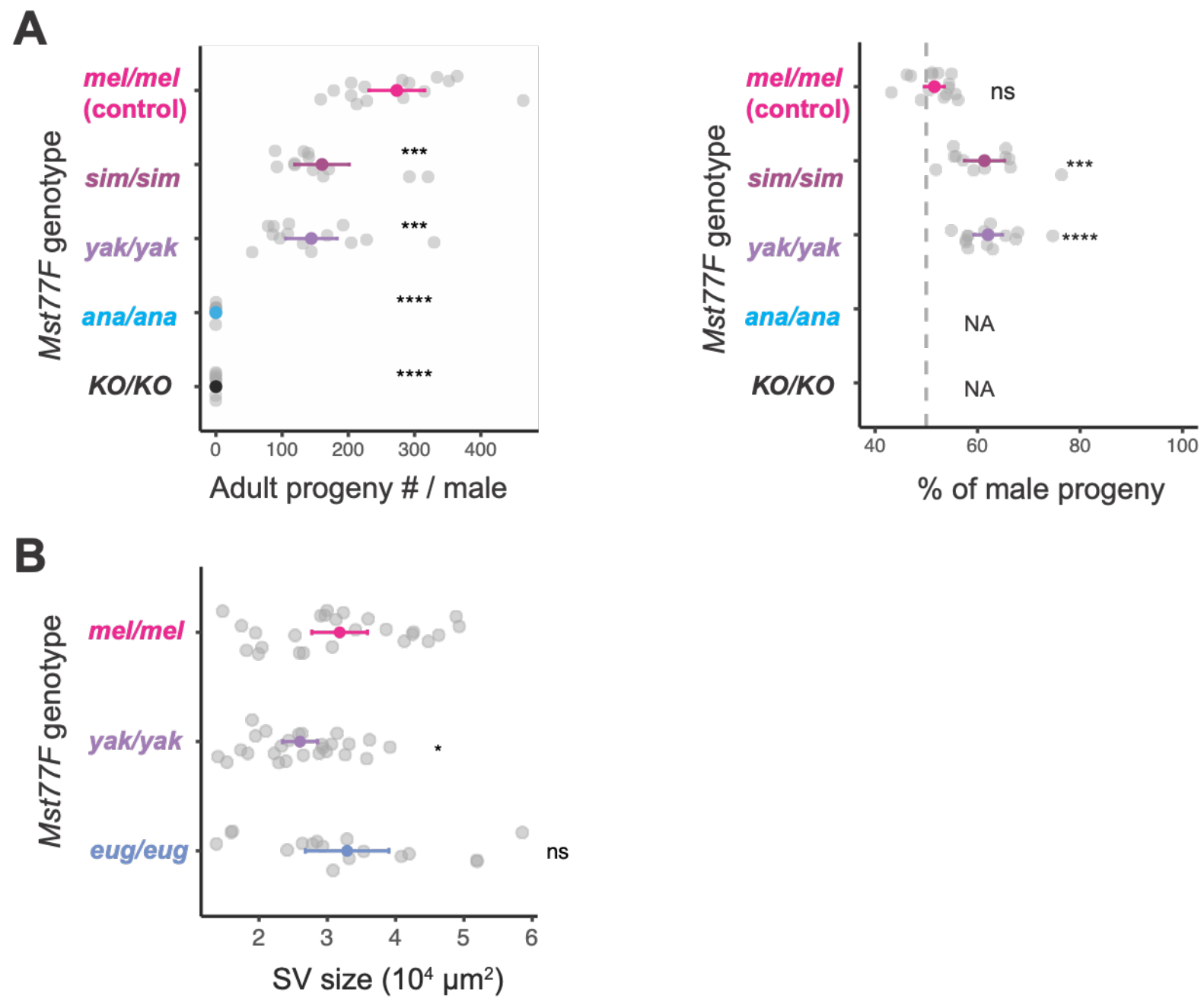

Fig. S4: *Mst77F* gene replacements in *D. melanogaster* reduce male fertility and result in sex-ratio distortion. **(A)** Similar to the result at 25°C, transgenic *D. melanogaster* males carrying two copies of *Mst77F-sim or Mst77F-yak* partially rescue fertility to levels and produce more male offspring comparable to *Mst77F-mel* at 29°C. In contrast, flies carrying two copies of the *Mst77F-ana* are sterile, just like knockout (*KO*) flies. **(B)** Transgenic males carrying *Mst77F-yak* exhibit reduced seminal vesicle size relative to *Mst77F-mel* controls, driven by a reduction in mature sperm quantity. Seminal vesicles in males carrying *Mst77F-eug* have comparable sizes relative to *Mst77F-mel* controls but display high size variability. All statistics are carried out using unpaired Student's *t*-tests against the control or χ^2 test against a 50:50 ratio (*p<0.05; **p<0.01; ***p<0.001; ****p < 0.0001; ns, not significant).

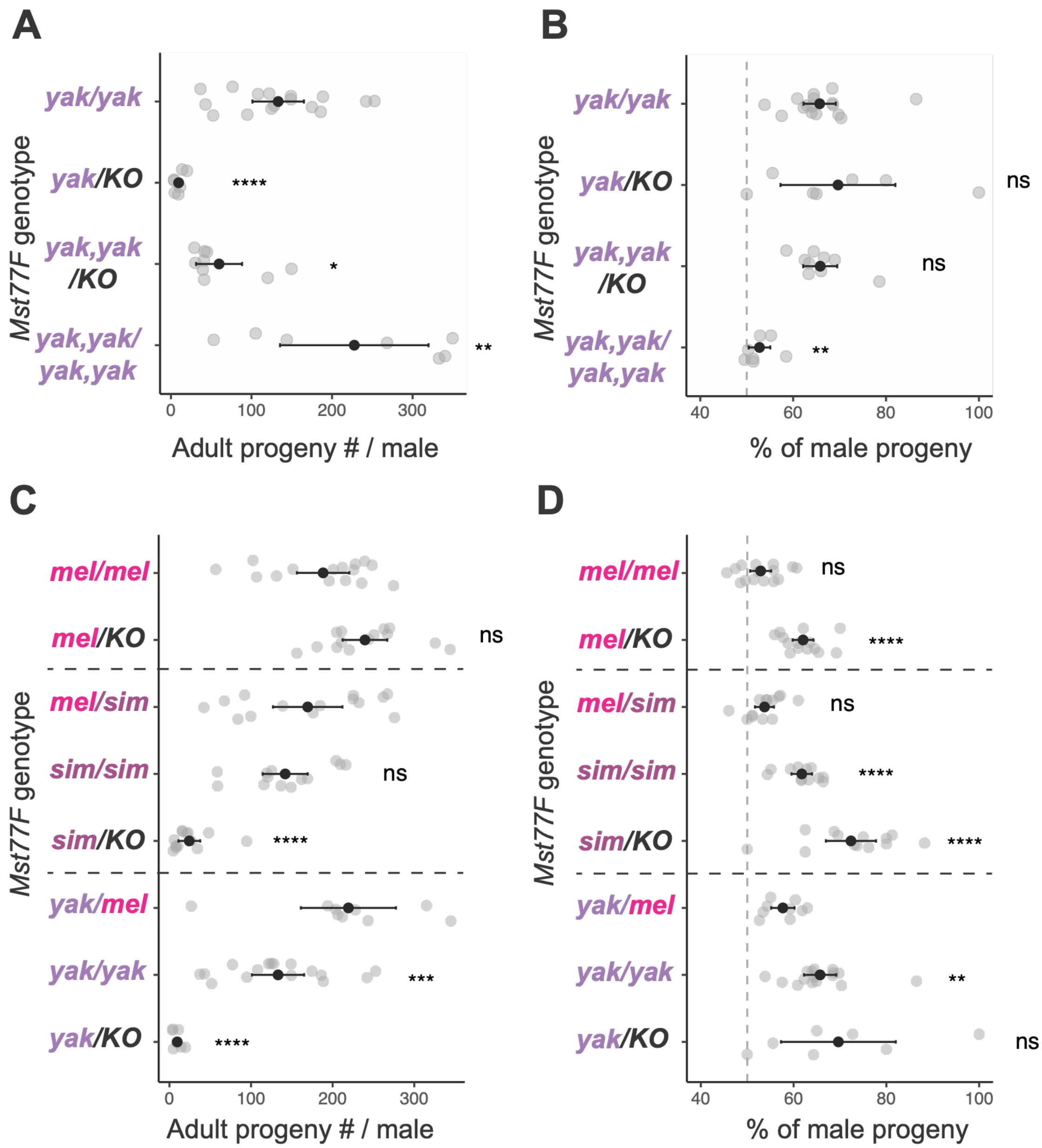

Fig. S5: Increased dosage or endogenous *Mst77F-mel* expression rescues fertility defects and sex-ratio distortion driven by *Mst77F* ortholog replacements. (A) Total adult progeny per male showing that supplementing with additional copies of *Mst77F-yak* rescues male fertility defects. **(B)** The percentage of male progeny from males carrying varying copy numbers of the *D. yakuba Mst77F* ortholog (*yak*) in an *Mst77F-mel* knockout (*KO*) background. An increased dosage of the *D. yakuba* transgene (*yak,yak/yak,yak*) significantly alleviates male-biased sex-ratio distortion toward the expected 50:50 baseline (indicated by the vertical dashed gray line). **(C)** Total adult progeny per male showing that supplementing with one additional copy of endogenous *Mst77F-mel* similarly restores male fertility. **(D)** The percentage of male progeny, demonstrating that the distorted male-biased sex ratios seen in hemizygous *Mst77F-sim* or *Mst77F-yak* males are alleviated by an additional copy of endogenous *Mst77F-mel*.

For all panels, horizontal dashed lines separate genetic groups and each dot represents data from a single male. Statistical comparisons for fertility **(A, C)** were evaluated using unpaired Student's *t*-tests, and sex ratios **(B, D)** were analyzed via χ^2 test against 50:50 ratio (*$p<0.05$; **$p<0.01$; ****$p < 0.0001$; ns, not significant).

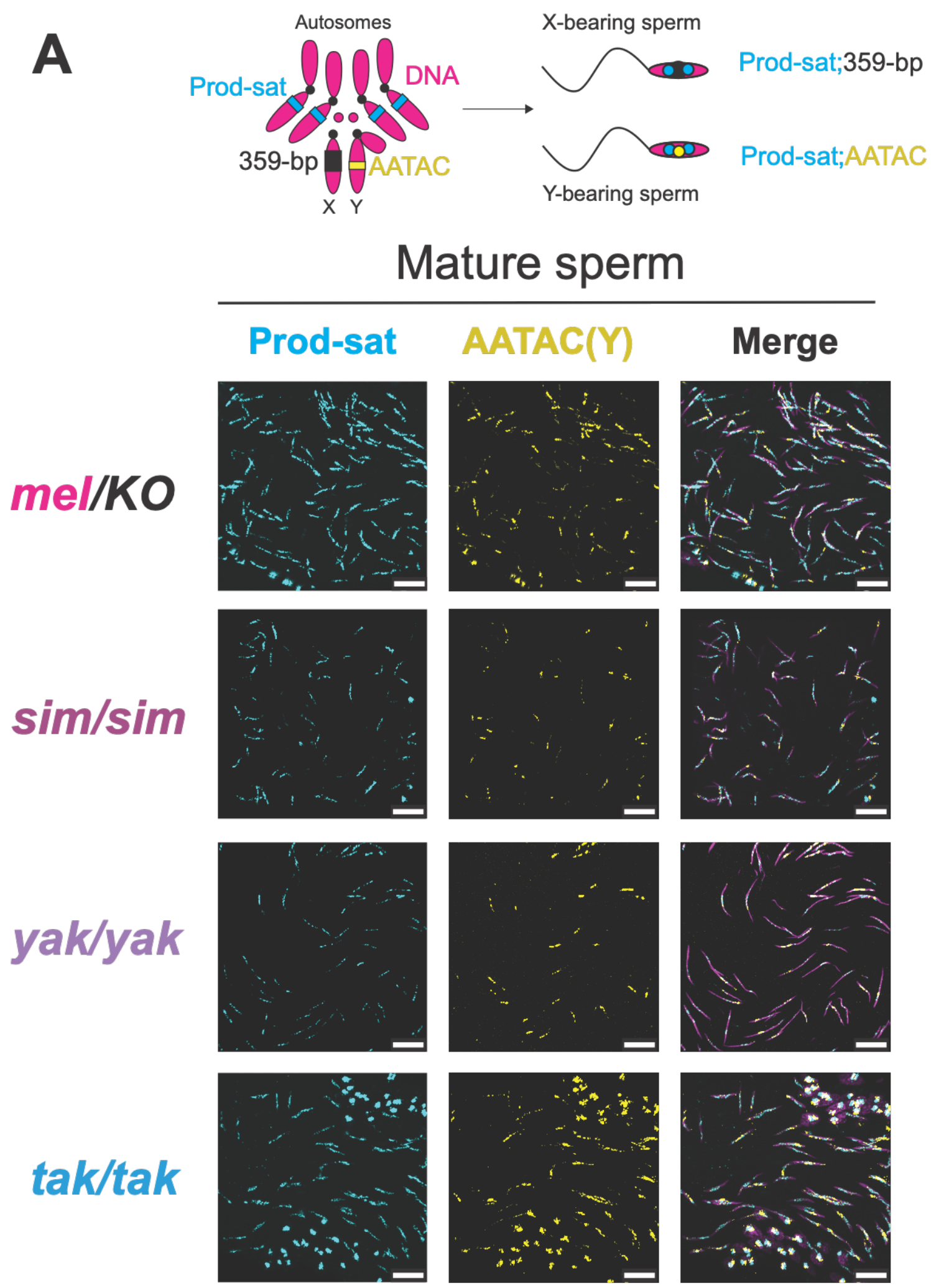

Fig. S6: *Mst77F* gene replacements in *D. melanogaster* decrease the proportion of X-bearing sperm. FISH was performed using the Y-linked AATAC (yellow) and Prod-sat (cyan) probes on seminal vesicles from homozygous *D. melanogaster* males carrying only one copy of *Mst77F-mel (mel/KO)* or two copies of *Mst77F* orthologs from other species (*sim/sim, yak/yak,* and *tak/tak)*.

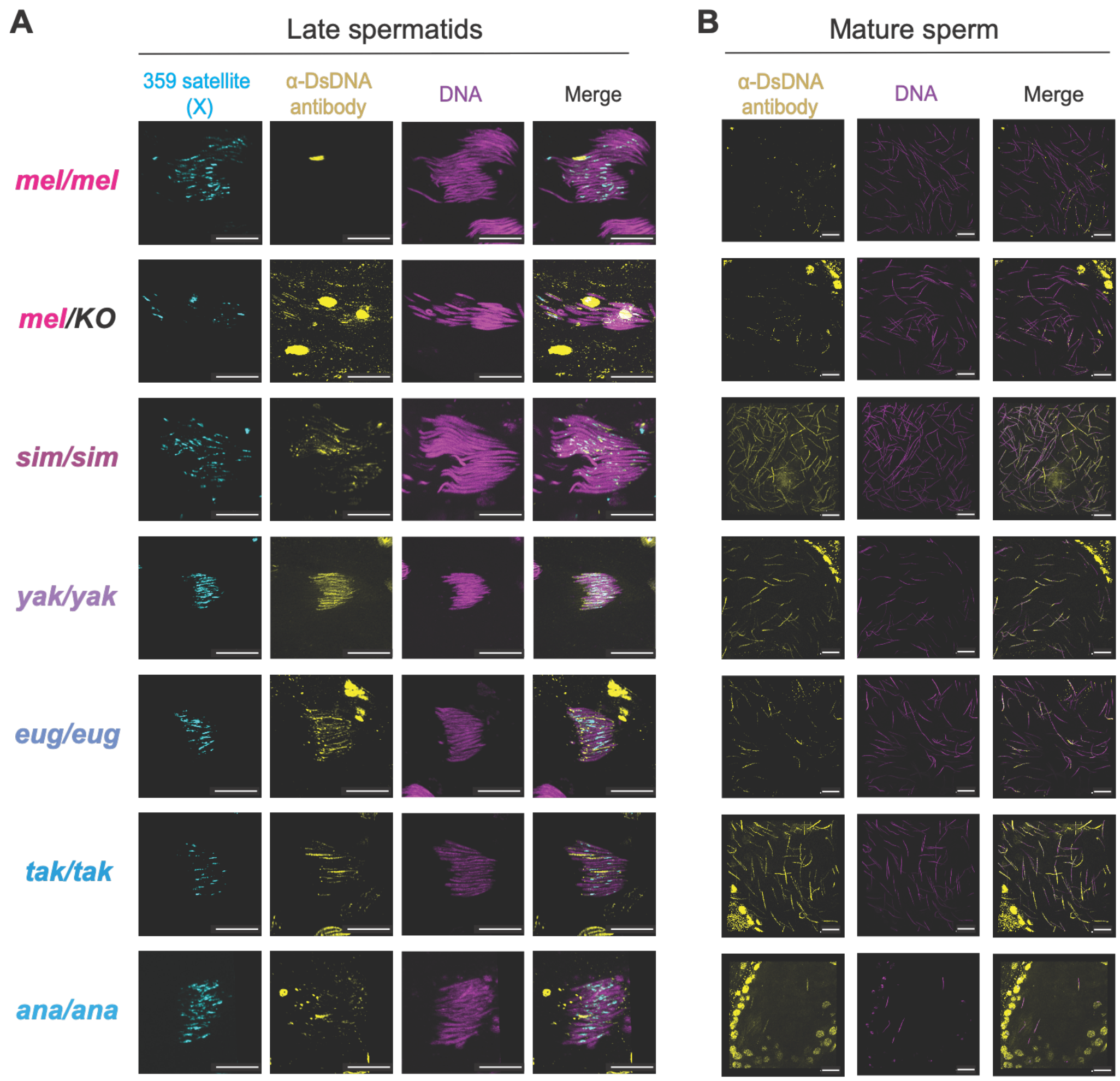

Fig. S7: *Mst77F* sequence divergence and dosage affect sperm nuclei condensation of X-bearing sperm in *D. melanogaster*. Late spermatids **(A)** and mature sperm **(B)** were stained with an α-dsDNA antibody (yellow) to assess their degree of chromatin compaction using immunostaining. DNA was stained with Hoechst 33342 (magenta). X-linked 359-bp satellite repeats were visualized with a DNA FISH probe (cyan). Late spermatids and mature sperm from flies carrying only one copy of *Mst77F-mel (mel/KO)* or two copies of *Mst77F* orthologs from other species (*sim/sim, yak/yak, eug/eug, tak/tak*, and *ana/ana)* exhibited chromatin compaction defects, as indicated by persistent α-dsDNA staining, whereas flies carrying two copies of *Mst77F-mel* (*mel/mel*) did not. In addition, flies with *Mst77F-ana/Mst77F-ana* have almost no mature sperm and are sterile. Scale bar = 10 μm for all images.

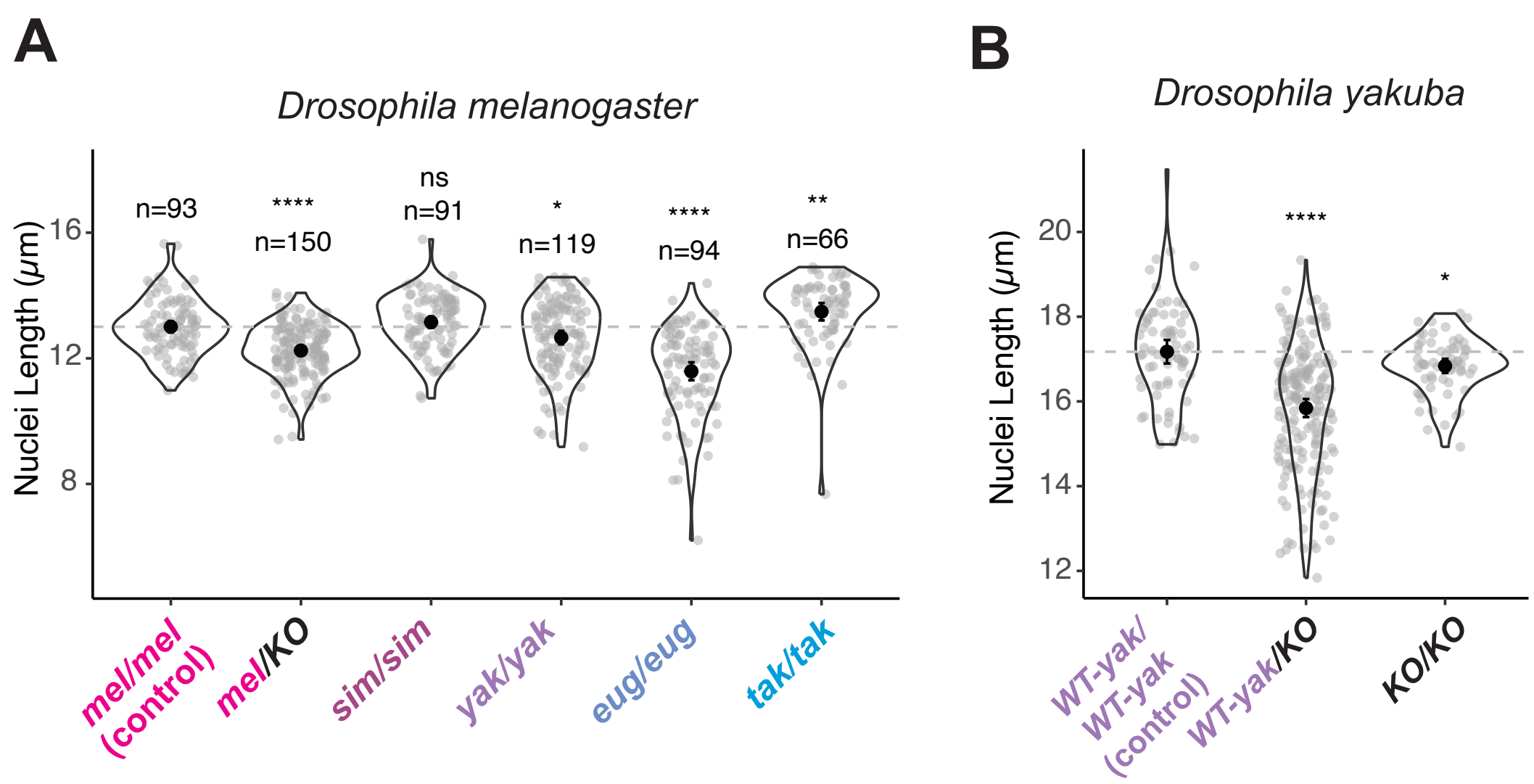

Fig. S8: *Mst77F* sequence divergence and dosage affect sperm nuclei length in *D. melanogaster*. (A) Sperm nuclei length (µm) in *D. melanogaster* males expressing different *Mst77F* orthologs. Sperm nuclei from transgenic flies with just one copy of *Mst77F-mel (mel/KO)* are shorter than those with two copies of *Mst77F-mel (mel/mel)*. *Mst77F-sim* homozygous males *(sim/sim)* do not have a significantly different sperm nuclear length than *Mst77F-mel* homozygotes. In contrast, *Mst77F-yak* or *Mst77F-eug* homozygous males *(yak/yak, eug/eug)* have reduced sperm nuclear length, whereas *Mst77F-tak* homozygous males *(tak/tak)* have more extended sperm nuclei. **(B)** Sperm nuclei from *D. yakuba* with no (*KO/KO)* or one copy of *Mst77F-yak (WT-yak/KO)* are shorter than those with two copies of WT-*Mst77F-yak (WT-yak/WT-yak)*. Statistical comparisons were performed using unpaired Student's *t*-tests (*p<0.05; **p<0.01; ****p < 0.0001; ns, not significant).

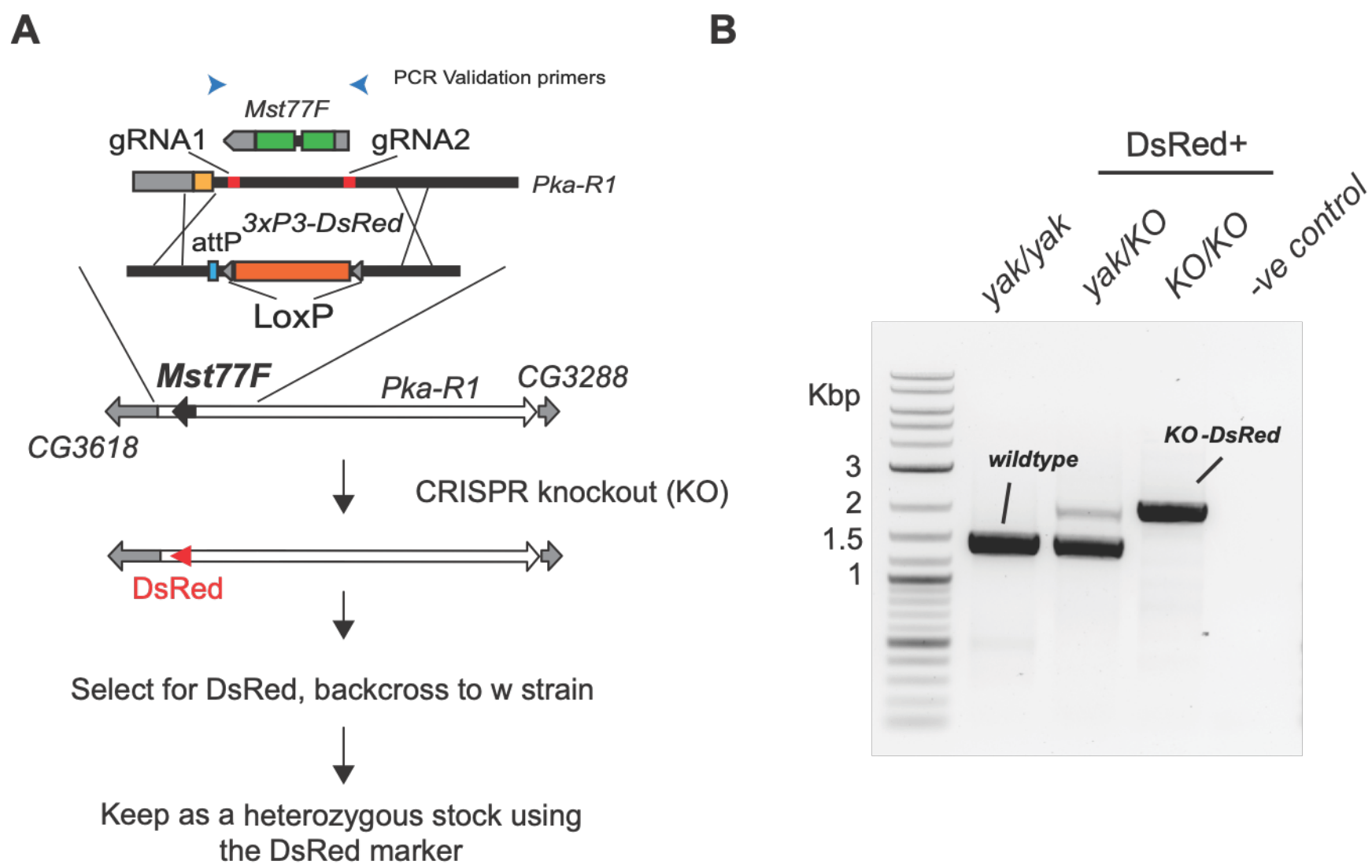

Fig. S9: Generation and validation of *Mst77F* knockouts in *Drosophila yakuba*. (A) Schematic illustrating the locations of guide RNA, gRNA1 and gRNA2, and a repair construct carrying a 3×P3-DsRed marker, an attP site and loxP sites. PCR validation primer sites are shown as blue arrowheads. *Mst77F-yak* knockout (*KO*) flies were generated using CRISPR-Cas9 in *D. yakuba*. We replaced the endogenous *Mst77F-yak* gene with *DsRed,* which was subsequently used for selection, strain establishment, and maintenance. Paternal genotype contribution was validated using offspring marker phenotypes *(e.g., DsRed* eye color). **(B)** PCR genotyping of flies carrying the *Mst77F-yak KO* reveals bands corresponding to the wild-type *Mst77F* and *KO* (*DsRed*) alleles in different genotypes, as well as a negative control.

Data S1. Primer sequences used in this study.

Data S2. Progeny counts from all the crosses.

Data S3. Quantification of Western blot analyses.

Data S4. Quantification of seminal vesicle sizes.

Data S5. Sperm counts from all genotypes.
